# Merged Tacrine-Based, Multitarget-Directed Acetylcholinesterase Inhibitors 2015–Present: Synthesis and Biological Activity

**DOI:** 10.3390/ijms21175965

**Published:** 2020-08-19

**Authors:** Todd J. Eckroat, Danielle L. Manross, Seth C. Cowan

**Affiliations:** School of Science, Penn State Erie, The Behrend College, Erie, PA 16563, USA; dmm6518@psu.edu (D.L.M.); svc5855@psu.edu (S.C.C.)

**Keywords:** acetylcholinesterase, tacrine, Alzheimer’s disease, multitarget-directed ligand, pyranopyrazole, Friedländer reaction

## Abstract

Acetylcholinesterase is an important biochemical enzyme in that it controls acetylcholine-mediated neuronal transmission in the central nervous system, contains a unique structure with two binding sites connected by a gorge region, and it has historically been the main pharmacological target for treatment of Alzheimer’s disease. Given the large projected increase in Alzheimer’s disease cases in the coming decades and its complex, multifactorial nature, new drugs that target multiple aspects of the disease at once are needed. Tacrine, the first acetylcholinesterase inhibitor used clinically but withdrawn due to hepatotoxicity concerns, remains an important starting point in research for the development of multitarget-directed acetylcholinesterase inhibitors. This review highlights tacrine-based, multitarget-directed acetylcholinesterase inhibitors published in the literature since 2015 with a specific focus on merged compounds (i.e., compounds where tacrine and a second pharmacophore show significant overlap in structure). The synthesis of these compounds from readily available starting materials is discussed, along with acetylcholinesterase inhibition data, relative to tacrine, and structure activity relationships. Where applicable, molecular modeling, to elucidate key enzyme-inhibitor interactions, and secondary biological activity is highlighted. Of the numerous compounds identified, there is a subset with promising preliminary screening results, which should inspire further development and future research in this field.

## 1. Introduction

**Figure 1:**
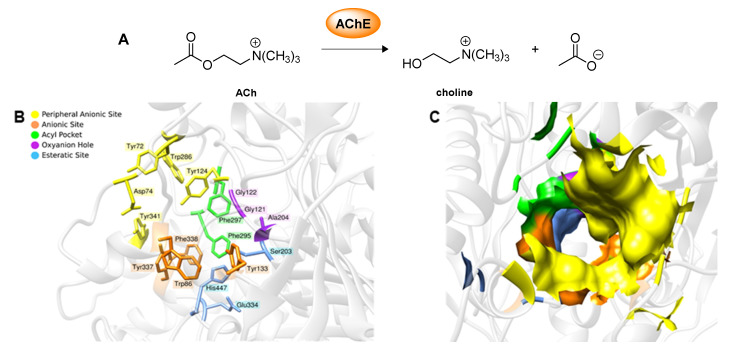
(**A**) Hydrolysis of acetylcholine (ACh) to choline and acetate catalyzed by acetylcholinesterase (AChE). (**B**) View of the *h*AChE (PDB:4EY4) active site with key amino acid residues color coded by region (reprinted from [3], with permission from Elsevier). (**C**) Surface representation of the *h*AChE (PDB:4EY4) active site looking down the gorge with regions color coded as in (**B**) (reprinted from [3], with permission from Elsevier).

Acetylcholinesterase (AChE; EC 3.1.1.7) catalyzes the hydrolysis reaction of acetylcholine (ACh) to choline and acetate (Figure 1A), making it responsible for terminating ACh-mediated synaptic transmission in the central nervous system (CNS) with high catalytic efficiency [1,2]. Structurally, three key features are known: The catalytic active site (CAS), the gorge, and the peripheral anionic site (PAS) (Figure 1B,C). The CAS is in the interior of the enzyme and is where the hydrolysis reaction takes place. It can be further divided into regions including the esteratic site, which contains the catalytic triad (Ser203, His447, Glu334 in *h*AChE (PDB:4EY4)), the anionic site, which contains aromatic residues (Trp86, Tyr133, Tyr337, Phe338) that stabilize the quaternary ammonium group of ACh through cation-π interactions, the oxyanion hole, which contains residues (Gly121, Gly122, Ala204) to stabilize the negatively charged transition state, and the acyl pocket, which uses residues (Phe295 and Phe297) to control substrate specificity. The gorge region, having dimensions of about 20 Å long and 5 Å wide, connects the CAS to the PAS and the enzyme exterior. It is lined with primarily aromatic amino acids. The PAS on the exterior of the enzyme also consists of primarily aromatic residues (Tyr72, Asp74, Tyr124, Trp286, Tyr341) and serves as a low affinity binding site to concentrate ACh at the entrance to the gorge [3,4,5,6,7,8,9].

**Figure 2:**
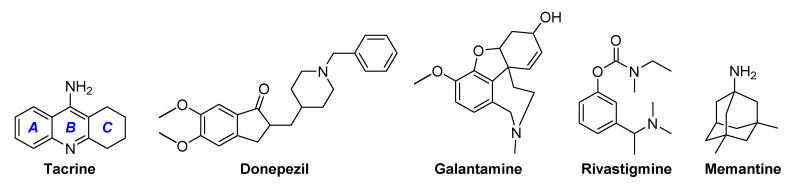
Structures of clinically used Alzheimer’s disease (AD) drugs. Tacrine (discontinued), donepezil, galantamine, and rivastigmine are all AChEi. Memantine is an *N*-methyl-d-aspartate receptor (NMDAR) antagonist. The tricyclic rings of tacrine are labeled *A–C* for discussion purposes.

AChE has received considerable attention as a target of chemical intervention, particularly for therapeutic treatment of Alzheimer’s disease (AD). In the United States, AD is the sixth leading cause of death and affects nearly 6 million people, and that number is estimated to grow to nearly 14 million in the next few decades. The disease is characterized by progressive neurodegeneration and diverse symptoms, the most noticeable being cognitive failure (memory loss, language difficulties, inability to plan or problem solve) [10]. The connection between AChE and AD dates back approximately 40 years and is based on the role of ACh in learning and memory and the observation of reduced cholinergic function in the brains of AD patients [11,12,13,14,15,16]. The logical progression has been to develop an AChE inhibitor(s) (AChEi) to slow the breakdown of ACh and boost its levels (cholinergic hypothesis). Indeed, over the last several decades, four AChEi (tacrine, donepezil, rivastigmine, and galantamine) have all been used to treat AD (Figure 2). A fifth drug used to treat AD, memantine, functions as an *N*-methyl-D-aspartate receptor (NMDAR) antagonist, and it is often used in combination with donepezil. While these drugs have had moderate success in alleviating cognitive symptoms, they are incapable of halting progression or curing AD [17]. This is thought to be, in large part, due to the complex nature and etiology of AD, and it is largely accepted that new and improved AD drugs should target additional factors beyond ACh.

As mentioned above, various other factors are thought to play a role in the AD onset and progression, and there is a significant amount of interplay among these factors and with AChE. The amyloid hypothesis focuses on extracellular aggregates of the amyloid-β (Aβ) peptide in the form of oligomers and plaques, which disrupt synaptic transmission and cause neuronal death. The Aβ peptide itself is generated by β-secretase (BACE1) and γ-secretase cleavage of the amyloid precursor protein (APP) [18,19,20,21,22]. Aβ aggregation is promoted through an interaction with the PAS of AChE [23,24,25] and with metal ions (Cu^2+^ and Zn^2+^) [26,27,28]. These metal ions may also, alone or in combination with Aβ oligomers, lead to the production of reactive oxygen species (ROS) and inflammation, which have been implicated in neuronal death and AD progression [29,30,31]. Dyshomeostasis of other intracellular metal ions, such as Ca^2+^, may also contribute to neurodegeneration [32,33]. Additionally, metabolic enzymes such as 15-lipoxygenase (15-LOX), which catalyzes the oxidation of polyunsaturated fatty acids to hydroperoxy acids, and cyclooxygenase-2 (COX-2), which is involved in the conversion of arachidonic acid to prostaglandins, contribute to ROS, oxidative stress, and inflammation [34,35,36,37]. Monoamine oxidase (MAO) is also of interest as the increase of monoaminergic neurotransmission through inhibition may alleviate AD symptoms, and ROS-byproducts of the MAO reaction exacerbate oxidative stress [38,39]. Carbonic anhydrase (CA) has even been implicated in AD, and modulation may be beneficial for AD [40,41]. Furthermore, the tau hypothesis focuses on abnormal phosphorylation of the tau protein. This protein is associated with microtubules and interacts with the neuronal cytoskeleton to facilitate intracellular signaling. When hyperphosphorylated, tau can aggregate to form intracellular neurofibrillary tangles (NFTs). This leads to compromised axonal transport and diminished synaptic function, and there is evidence that tau may act synergistically with Aβ [18,42]. Finally, the mitochondrial cascade hypothesis of AD suggests that the individual genetic makeup determines a baseline mitochondrial function, and the genetics plus environment determines how the mitochondrial function declines with age. The declining mitochondrial function can then initiate other pathologies associated with AD, such as Aβ aggregation and tau phosphorylation [43,44].

Tacrine was the first AChEi approved by the United States Food and Drug Administration for the treatment of AD in 1993. However, it is no longer used clinically due to poor pharmacokinetics requiring four times per day dosing and side effects, the most notorious being hepatotoxicity. Structurally, it is a tricyclic molecule (see Figure 2) consisting of an aromatic *A*-ring, heteroaromatic *B*-ring containing an amino substituent at position 9, and a saturated *C*-ring. Hepatotoxicity has been linked to *A*-ring hydroxylation during metabolism, which is followed by subsequent conversion to a reactive quinone-like metabolite [45,46,47,48]. Tacrine has, however, remained an important molecule for research into new AD drugs. This is largely due to its strong inhibition of AChE (low nanomolar IC_50_), ligand efficiency, synthetic accessibility, tolerance of structural modification, and suitability as a starting point for the multitarget-directed ligand (MTDL) strategy. As mentioned above, AD is multifactorial in nature and new and improved AD drugs should target additional factors beyond the cholinergic system. The MTDL strategy for AD, which aims to combine two or more pharmacophores into a single chemical entity capable of acting on multiple aspects of AD at once, has been implemented using tacrine with varying degrees of success for nearly three decades [49,50,51,52,53,54]. It should be noted that the MTDL strategy is not unique to AD. For example, it has been applied to the treatment of cancer, malaria, and diabetes [55,56].

**Figure 3:**
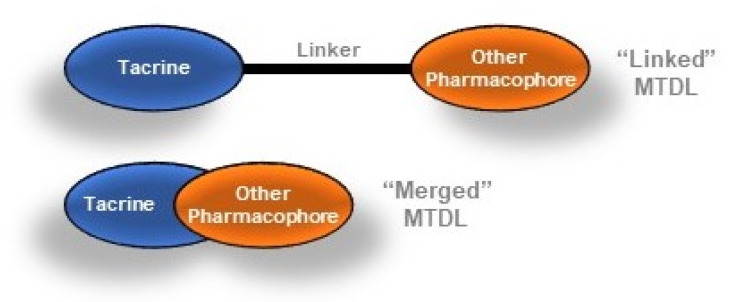
Tacrine-based multitarget-directed ligand (MTDLs) can be classified as “linked” or “merged” based on the degree of overlap of the pharmacophores (adapted with permission from [57], copyright (2005) American Chemical Society).

Morphy and Rankovic [57] classified MTDLs based on the degree of overlap of the pharmacophores (Figure 3). “Linked” MTDLs contain well separated pharmacophores joined by a distinct linker region that is not present in either parent compound. “Merged” MTDLs represent the opposite end of a continuum where there is a high degree of overlap between the pharmacophores based on structural similarities in the parent compounds. The merged approach often results in smaller and simpler molecules, whereas the linked approach leads to larger, higher molecular weight molecules. The tacrine-based MTDLs reported in the literature are numerous, and our initial survey led us to divide tacrine-based MTDLs into two groups: Linked and merged. To keep the scope of this manuscript manageable, we report herein only on the merged MTDLs. Furthermore, to focus on current advances, this review details only compounds that were newly reported in the last five years (2015–present). While we recognize the significance of linked tacrine-based MTDLs to this field, we feel that a detailed discussion warrants a separate report, which will be composed in due course.

The merged tacrine-based MTDLs discussed have been grouped based on structural similarities. Special emphasis is placed on the synthesis of the compounds and comparison of in vitro AChE inhibition to tacrine, 6-chlorotacrine, or 7-methoxytacrine as appropriate. The 6-chloro substituent on the tacrine scaffold is known to improve the inhibition of AChE while increasing toxicity, and the 7-methoxy substituent, conversely, is known to weaken inhibition while reducing toxicity [58,59]. Unless otherwise stated, inhibition is evaluated using the classic Ellman method [60] with AChE from either *Electrophorus electricus* (*Ee*AChE) or human (*h*AChE). These two enzymes have a 56.6% sequence identity, but *Ee*AChE is generally the less expensive choice for preliminary testing [61]. We highlight key structure-activity relationship (SAR) trends and in silico molecular modeling interactions where appropriate, while also describing relevant secondary in vitro and in vivo biological activity. Molecular modeling uses either *h*AChE or *Torpedo californica* AChE (*Tc*AChE), which have a 51.9% sequence identity [61]. Together, the in vitro, in silico, and in vivo data represent an “in combo” approach to AD drug discovery [56,62].

## 2. Pyranopyrazole Tacrines

**Scheme 1:**
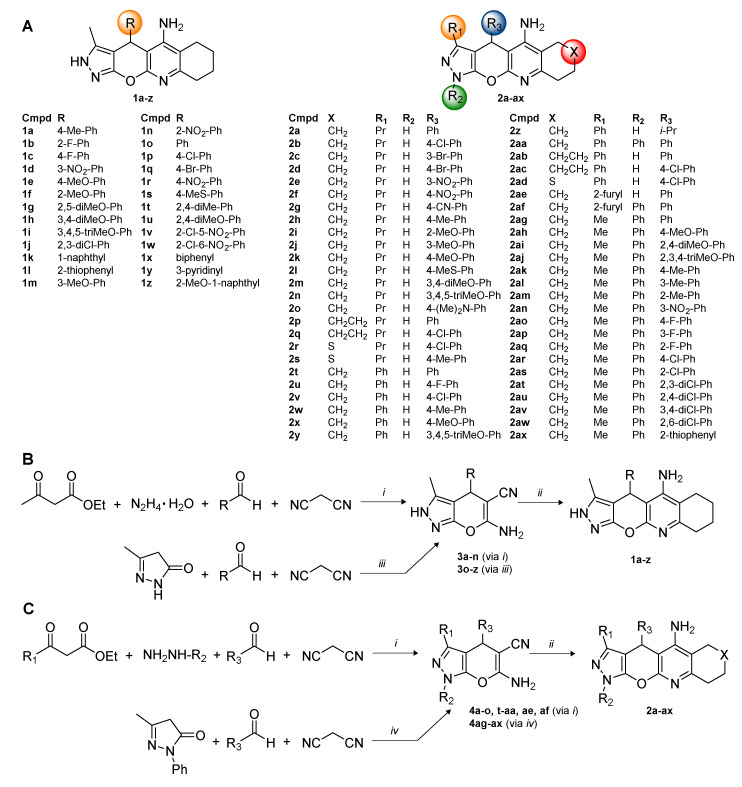
(**A**) Structure of pyranopyrazole tacrines **1a**–**z** and **2a**–**ax** [63,64,65,66,67]. (**B**) Synthesis of pyranopyrazole tacrines **1a**–**z**. (**C**) Synthesis of pyranopyrazole tacrines **2a**–**ax**. Reagents and conditions: (*i*) Ultrasonic irradiation, (*S*)-Pro, H_2_O/EtOH, rt, 10–35 min, 77–95%; (*ii*) cyclohexanone or cycloheptanone or tetrahydro-4H-thiopyran-4-one, AlCl_3_, DCE or DCM, Δ (12–24 h, 65–95%) or MWI (53–90%); (*iii*) DCE, Δ, 3–4 h, quant.; (*iv*) DABCO, EtOH, rt, 12 h, quant.

One of the most studied modifications to the tacrine core over the last five years has been the replacement of the aromatic *A*-ring with a fused pyranopyrazole moiety. The resulting 76 different analogs of this tetracyclic scaffold can be divided among two series: **1a**–**z**, varying in the 4-aryl substituent on the pyran ring, and **2a**–**ax**, varying at four positions around the scaffold (Scheme 1A) [63,64,65,66,67]. Synthetically, pyranopyrazole tacrines are accessed via closely related two-step sequences (Scheme 1B,C). The pyrano[2,3-*c*]pyrazole core can be constructed using a one-pot four-component reaction between β-ketoesters, hydrazine hydrate or hydrazine derivatives, malononitrile, and aryl or alkyl aldehydes under ultrasonic irradiation in the presence of (*S*)-Pro to give **3a**–**n** or **4a**–**o**, **t**–**aa**, **ae**, **af** [63,64,66]. Alternatively, the pyrano[2,3-*c*]pyrazole core **3o**–**z** or **4ag**–**ax** can be constructed from the reaction of 3-methyl-1*H*-pyrazol-5(4*H*)-one or 3-methyl-1-phenyl-1*H*-pyrazol-5(4*H*)-one with aryl aldehydes and malononitrile [65,67]. A subsequent Friedländer reaction with the appropriate cyclohexanone, cycloheptanone, or tetrahydro-4*H*-thiopyran-4-one and AlCl_3_ gave the target compounds **1a**–**z** and **2a**–**ax** as racemic mixtures.

In addition to acting as AChEi, pyranopyrazole tacrines have shown activity against ROS and Aβ. Among compounds reported by Khoobi et al., the most active inhibitor **1h** (*Ee*AChE IC_50_ = 190 nM) showed a slight improvement over tacrine (*Ee*AChE IC_50_ = 280 nM) (Table 1). SAR showed that a methoxy substituent at the 4-position of the phenyl ring showed better inhibition than methyl or fluoro substituents, and a second methoxy group at the 3-position, as seen in **1h**, further improved potency. The Lineweaver-Burk plot showed varying x- and y-intercepts indicating a mixed-type inhibition for this compound (i.e., binding to both the substrate active site (CAS) and a second distinct site (PAS)). Molecular modeling predicted the *R*-enantiomer of **1h** to interact with *Tc*AChE near the CAS. The methoxyphenyl group showed a key hydrogen bonding interaction with His440 of the catalytic triad. However, the *S*-enantiomer was predicted to interact with the top of the gorge near the PAS. At 10 µM, **1h** showed similar neuroprotection to quercetin in response to H_2_O_2_-induced damage in PC12 cells [63]. Chioua et al. identified two promising *Ee*AChEi in the 4-nitrophenyl analog **1r** (IC_50_ = 170 nM), which was comparable to tacrine (IC_50_ = 190 nM), and the 2-methoxyphenyl analog **1f** (IC_50_ = 1.52 µM), which was 8-fold less potent than tacrine (Table 1). Interestingly, the Lineweaver-Burk plot indicated that **1f** was a noncompetitive inhibitor, while molecular modeling of (*R*)- and (*S*)-**1r** with *Ee*AChE showed similar interactions confined to the PAS, namely π-π stacking interactions between the pyranopyrazole moiety and Trp286 and the pyridine moiety and Tyr341. Furthermore, both **1f** and **1r** showed complete inhibition of Aβ_1-40_ *Ee*AChE-induced aggregation at 25 µM, and they showed comparable neuroprotection against oligomycin A/rotenone-induced oxidative stress in cortical neurons. However, only **1f** was nontoxic to HepG2 cells at 300 µM and showed favorable in silico CNS permeability [64]. Compounds **1s**, with a para-thioanisole substituent, and **1x**, with a biphenyl substituent, were also highly potent *Ee*AChEi being 4.5- to 6-fold more potent than tacrine and having IC_50_ = 58 and 44 nM, respectively (Table 1). Molecular modeling indicated that both enantiomers of **1x** interacted with *Tc*AChE at the gorge entrance with the biphenyl substituent positioned towards the CAS [65].

**Figure 4:**
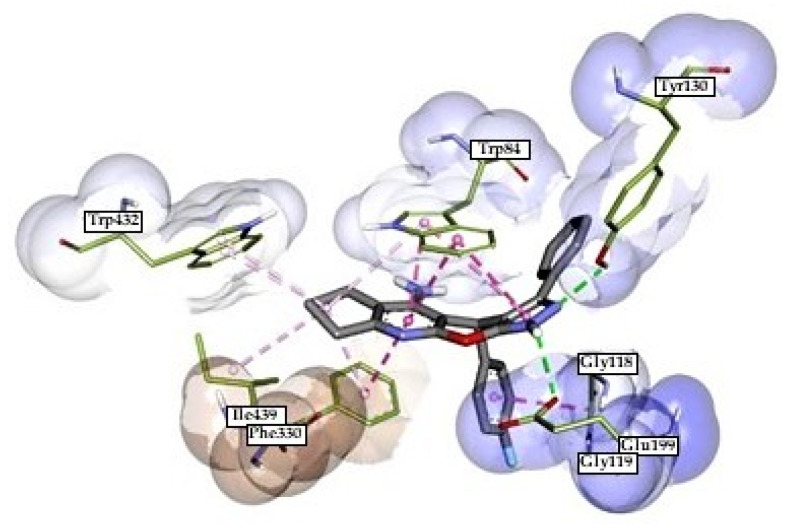
Molecular modeling of **2u** in the substrate active site (CAS) of *Tc*AChE (adapted from [66], with permission from Elsevier). The pyrazole ring forms key H-bonds with Glu199 and Tyr130 and π-π stacking with Trp84. The 4-fluorophenyl moiety forms an amide-π stacking interaction with Gly119.

Additional studies have focused on varying ring size on the tacrine core and side chains off of the pyrano[2,3-*c*]pyrazole moiety [66,67]. Compounds **2a**–**af**, with one exception, all showed IC_50_ < 2 µM (*Ee*AChE). SAR results showed that a propyl or phenyl substituent at R_1_ and a 4-methoxyphenyl or 4-fluorophenyl substituent at R_3_ gave the best inhibition. Expansion of the tacrine cyclohexane to a cycloheptane gave mixed inhibition results, and bioisosteric replacement of a cyclic methylene with S had little effect. Of note, compared to tacrine (IC_50_ = 221 nM), **2u** (IC_50_ = 34 nM) and **2x** (IC_50_ = 81 nM) were 6- and 3-fold more potent, respectively. Molecular modeling of **2u** with *Tc*AChE (Figure 4) showed the pyrazole ring forming key H-bonds with Glu199 and Tyr130 and π-π stacking with Trp84 in the CAS. Additionally, the 4-fluorophenyl moiety was positioned towards the oxyanion hole forming an amide-π stacking interaction with Gly119. The Lineweaver-Burk plot showed a mixed-type inhibition for **2u**. Moreover, **2x** was a moderate inhibitor of 15-LOX (IC_50_ = 31 µM, compared to quercetin IC_50_ = 18 µM), while **2u** showed no inhibition of this enzyme, and both **2u** and **2x** were less hepatotoxic than tacrine, showing no appreciable change in the HepG2 cell viability up to 50 µM [66]. Compounds **2ag**–**ax** were overall less potent inhibitors of AChE. Of this series, **2ag** and **2ah** with a phenyl and 4-methoxyphenyl substituent off the pyran, respectively, showed the best inhibition of *Ee*AChE with IC_50_ ~ 2.8 µM [67].

## 3. Pyranopyranone Tacrines

**Scheme 2:**
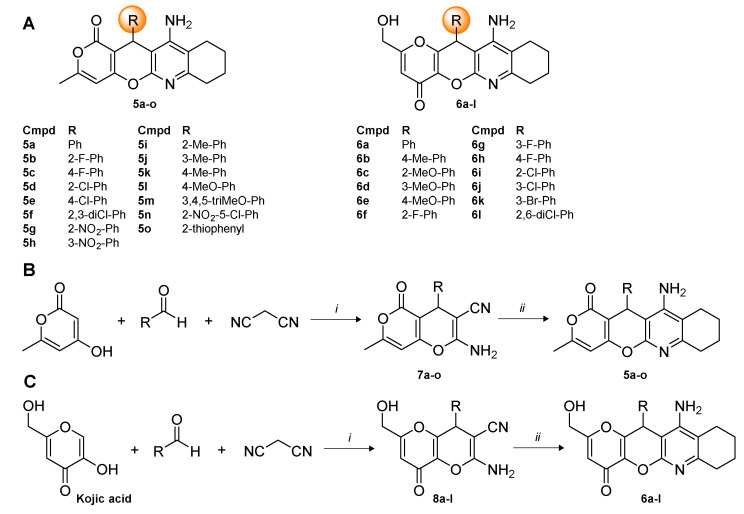
(**A**) Structure of pyranopyranone tacrines **5a**–**o** and **6a**–**l** [68,69]. (**B**) Synthesis of pyranopyranone tacrines **5a**–**o**. (**C**) Synthesis of pyranopyranone tacrines **6a**–**l**. Reagents and conditions: (*i*) DABCO (rt, 12 h, quant.) or Et_3_N (Δ, 5 min, 63–77%), EtOH, rt; (*ii*) cyclohexanone, AlCl_3_, DCE or 1,4-dioxane, Δ, 2–18 h, 50–87%.

There have also been recent reports of pyranopyranone tacrines **5a**–**o** and **6a**–**l** (Scheme 2A) [68,69]. These compounds contain hydroxypyranone moieties that offer activity against ROS, while maintaining a potent inhibition of AChE. The target compounds were prepared in two steps from a starting hydroxypyranone (Scheme 2B,C). 4-Hydroxy-6-methyl-2*H*-pyran-2-one or kojic acid (KA), a natural fungal metabolite with ROS scavenging ability [70], were reacted with aryl aldehydes and malononitrile in the presence of DABCO or Et_3_N to afford intermediate aminocarbonitriles **7a**–**o** and **8a**–**l**. Then, the Friedländer reaction with cyclohexanone in the presence of AlCl_3_ gave the target compounds **5a**–**o** and **6a**–**l** as racemic mixtures [68,69].

**Figure 5:**
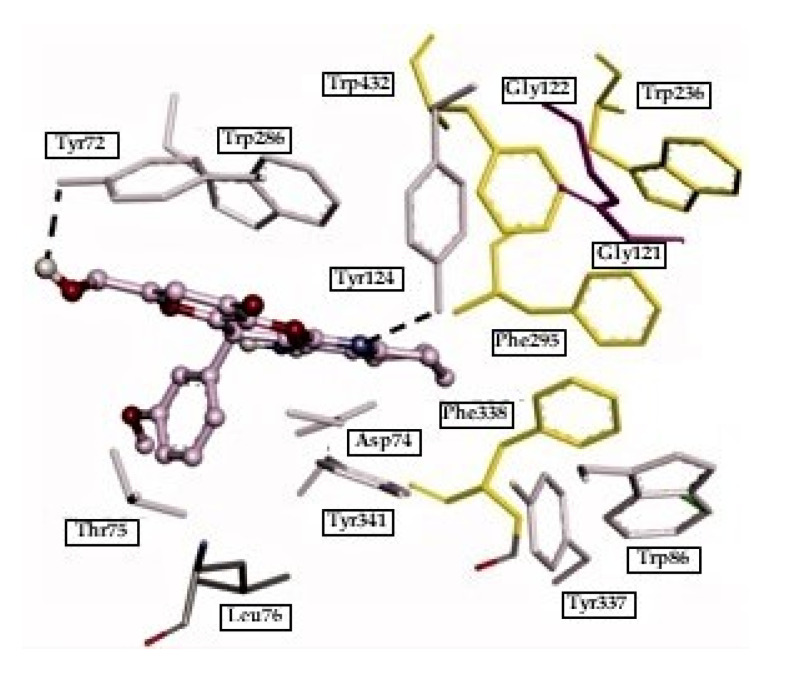
Molecular modeling of (*R*)-**6d** in the second distinct site (PAS) of *h*AChE (adapted from [69]). Important interactions include π-π stacking with Trp286 and Tyr341 and H-bonds of the primary hydroxyl group with Tyr72 and the tacrine-like amino group with Tyr124.

Compared to tacrine (*Ee*AChE IC_50_ = 48 nM), **5a**–**o** showed a weaker inhibition. The most potent compound **5f** with a 2,3-dichlorophenyl group was 8-fold weaker (*Ee*AChE IC_50_ = 370 nm). **5f** also showed moderate neuroprotection in PC12 cells against H_2_O_2_-induced damage, but it did not perform as well as quercetin in the same assay. The Lineweaver-Burk plot of **5f** showed a mixed inhibition, suggesting an interaction with both the CAS and PAS. Interestingly, molecular modeling showed that the *R*-enantiomer of **5f** was predicted to interact with the PAS, while the *S*-enantiomer was predicted to interact with the mid-gorge region and span the PAS-CAS distance [68]. Similarly, **6a**–**l** also showed a comparatively weaker inhibition. Of this series, the most potent inhibitor of *Ee*AChE was the 3-methoxyphenyl substituted **6d** (IC_50_ = 640 nM), which was 20-fold less potent than tacrine (IC_50_ = 31 nM). Promisingly though, most of these compounds were significantly less toxic than tacrine. In addition, **6d** maintained a 65.5% viability in HepG2 cells at 1 mM, 6-fold less toxic than tacrine (10.9% viability at 1 mM). Moreover, **6d** improved upon the known antioxidant capacity of KA (ORAC assay; 4.79 and 2.51 Trolox equivalents for **6d** and KA, respectively), and it showed a capable neuroprotection against oligomycin/rotenone and Aβ_1-40_ in SH-SY5Ycells at 3 µM. Molecular modeling of *h*AChE with **6d** *R*- and *S*-enantiomers indicated an association of both with the PAS via π-π stacking with Trp286 and Tyr341, acting as a barrier to the active site of the enzyme (Figure 5). Additional key H-bonds were noted, including the primary hydroxyl group for both enantiomers with Tyr72 and the tacrine-like amino group with Tyr124 (*R*-enantiomer) or Asp74 (*S*-enantiomer) [69].

## 4. Pyranonaphthalene, Pyranoquinoline, and Pyranonaphthoquinone Tacrines

**Scheme 3:**
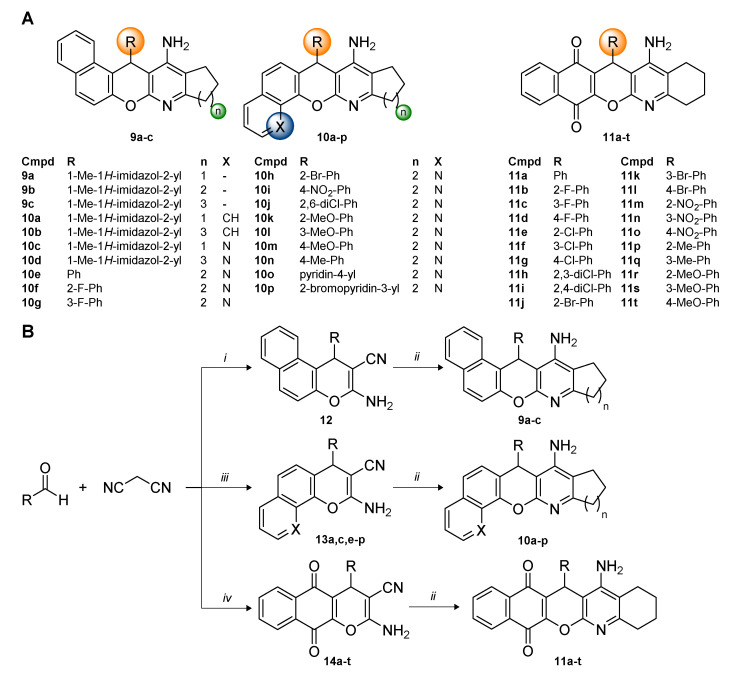
(**A**) Structure of pyranonaphthalene (**9a**–**c**, **10a,b**), pyranoquinoline (**10c**–**p**), and pyranonaphthoquinone tacrines **11a**–**t** [71,72,73,74]. (**B**) Synthesis of pyranonaphthalene (**9a**–**c**, **10a,b**), pyranoquinoline (**10c**–**p**), and pyranonaphthoquinone tacrines **11a**–**t**. Reagents and conditions: (*i*) 2-Naphthol, piperidine, EtOH, Δ, 12 h, 72%; (*ii*) cyclopentanone or cyclohexanone or cycloheptanone, AlCl_3_, DCE or 1,4-dioxane, Δ (2–24 h, 40–95%) or MWI (3 h, 9–70%); (*iii*) 1-naphthol or 8-hydroxyquinoline, piperidine, EtOH, Δ, 15 min–12 h, 47–93%; (*iv*) 2-hydroxy-1,4-naphthoquinone, piperidine, EtOH, Δ, 2–6 h, quant.

Pentacylcic pyranotacrines bearing a fused naphthalene, quinoline, or naphthoquinone moiety (Scheme 3A) have shown diverse anti-AD activity, including AChE inhibition, BACE1 inhibition, ROS scavenging, and metal chelation [71,72,73,74]. The pyranoquinoline tacrines were designed to incorporate the hydroxyquinoline moiety of cliquinol, a known antioxidant and Cu-chelator [72,75], while the pyranonaphthoquinone tacrines were designed to incorporate the known 1,4-naphthoquinone BACE1i scaffold [73,76]. The compounds were prepared through closely related two-step sequences relying on 4*H*-pyran construction from malononitrile, aryl aldehydes, and either 2-naphthol, 1-naphthol, 8-hydroxyquinoline, or 2-hydroxy-1,4-naphthoquinone to give intermediates **12** [71], **13a**,**c**,**e**–**p** [71,72], and **14a**–**t** [73,74] (Scheme 3B). In all cases, a subsequent Friedländer reaction with the appropriate cycloalkanone and AlCl_3_ gave the target compounds **9a**–**c**, **10a**–**p**, and **11a**–**t** as racemic mixtures.

In terms of biological activity, these compounds showed a weak inhibition of AChE with varying secondary activity. Among pyranonaphthalene and pyranoquinoline tacrines bearing a 1-methyl-1*H*-imadazol-2-yl substituent **9a**–**c** and **10a**–**d**, compared to tacrine (*Ee*AChE IC_50_ = 89.8 nM), all were weaker inhibitors of AChE. Even the most potent inhibitor **9b** (*Ee*AChE IC_50_ = 6.73 µM) was 75-fold less potent than tacrine. Non-competitive inhibition was indicated by the Lineweaver-Burk plot and confirmed by molecular modeling, which showed both enantiomers of **9b** favoring an interaction with the PAS. The antioxidant capacity was promising, however, with compounds exhibiting between 1.47–2.75 Trolox equivalents by the ORAC assay, but **9b** did show increased hepatotoxicity compared to tacrine in HepG2 cells [71]. Among **10e**–**p**, **10l** bearing a 3-methoxyphenyl substituent was the most potent against *Ee*AChE (IC_50_ = 40 nM, compared to tacrine IC_50_ = 30 nM). SAR indicated the phenyl substituted inhibitors to be more potent than the pyridinyl substituted ones. The Lineweaver-Burk plot showed that **10l** acted as a noncompetitive inhibitor of *h*AChE. Molecular modeling predicted that the *R*-enantiomer bound to *h*AChE at the PAS primarily through π-π stacking interactions. Moreover, noted were two H-bonds between the 8-amino group and Asp74 and Leu76. The *S*-enantiomer was predicted to bind in a similar fashion. These compounds showed a moderate antioxidant ability, and **10l**, although not the best antioxidant tested, gave an ORAC assay result of 1.83 Trolox equivalents. In addition, **10l** was non-toxic in HepG2 cells at concentrations up to 1 mM and was predicted to cross the blood-brain barrier (BBB) by the parallel artificial membrane permeability assay (PAMPA) (effective permeability (*P_e_*) = 5.41 × 10^−6^ cm/s where a high BBB permeation predicted with *P_e_* (10^−6^ cm/s) > 4.0, a low BBB permeation predicted with *P_e_* (10^−6^ cm/s) < 2.0, and BBB permeation uncertain with *P_e_* (10^−6^ cm/s) from 2.0 to 4.0). Disappointingly, **10l** showed not ability to chelate Cu^2+^ or Fe^2+^ [72]. The pyranonaphthoquinone tacrines **11a**–**t** showed significantly less inhibition of AChE than tacrine. The 3-nitrophenyl substituted **11n** (*Ee*AChE IC_50_ = 860 nM) was 17-fold less potent than tacrine (*Ee*AChE IC_50_ = 50 nM) [73], and the 4-methoxyphenyl substituted **11t** (*h*AChE IC_50_ = 1.10 µM) was 8.5-fold less potent than tacrine (*h*AChE IC_50_ = 130 nM) [74]. **11n** and **11t** showed a mixed-type inhibition by the Lineweaver-Burk plot analysis, while molecular modeling with *Tc*AChE showed a similar interaction for both compounds with CAS and PAS residues. Key interactions included π-π interactions with the aromatic rings and Phe330 and Trp84 in the CAS, as well as an H-bond between the nitro substituent (for **11n**) and Tyr334 in the PAS [73,74]. Additionally, **11n** showed a promising ability to chelate Cu^2+^, Zn^2+^, and Fe^2+^. Unfortunately though, **11n** showed only a weak BACE1 inhibition compared to the known peptidomimetic BACE1 inhibitor OM99-2 (IC_50_ = 19.60 µM and 14 nM, respectively), and it showed no antioxidant activity or neuroprotective ability against Aβ_25-35_ [73]. Importantly, **11t** showed 3.5-fold less hepatotoxicity than tacrine at 1 mM in HepG2 cells and was predicted to be CNS active (PAMPA-BBB, *P_e_* = 4.4 × 10^−6^ cm/s) [74].

## 5. Other Pyranotacrines

**Scheme 4:**
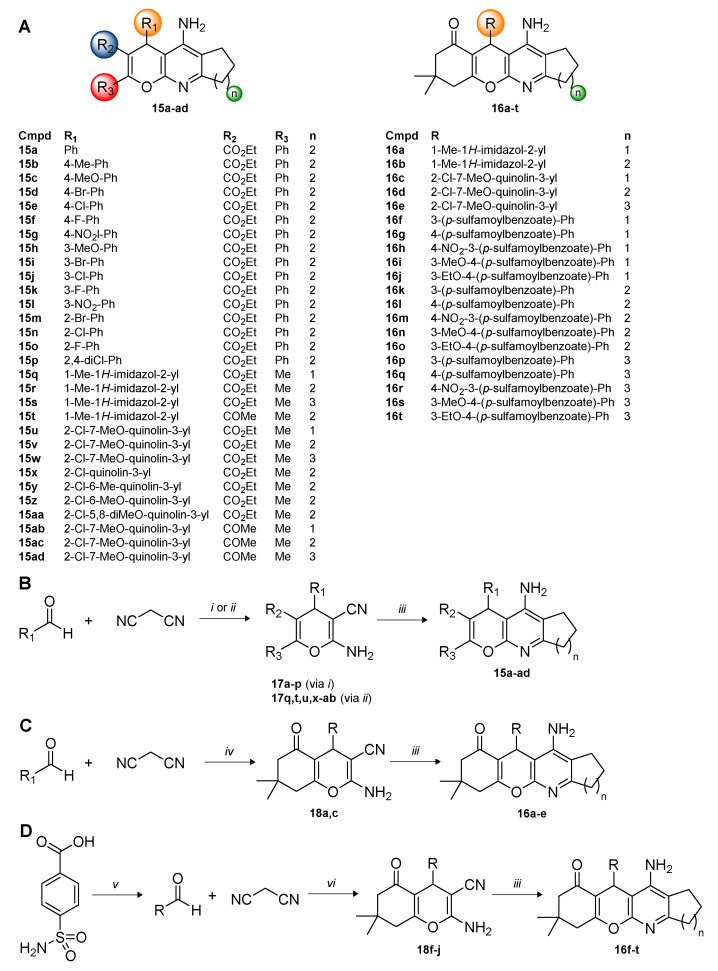
(**A**) Structure of other pyranotacrines **15a**–**ad** and **16a**–**t** [71,77,78,79]. (**B**) Synthesis of pyranotacrines **15a**–**ad**. (**C**) Synthesis of pyranotacrines **16a**–**e**. (**D**) Synthesis of sulfamoyl-containing pyranotacrines **16f**–**t**. Reagents and conditions: (*i*) Ethyl benzoylacetate (R_2_ = CO_2_Et, R_3_ = Ph), piperidine, EtOH, Δ, 3 h, quant.; (*ii*) ethyl acetoacetate (R_2_ = CO_2_Et, R_3_ = Me) or acetylacetone (R_2_ = COMe, R_3_ = Me), piperidine, EtOH, rt, 12 h, 78–93%; (*iii*) cyclopentanone or cyclohexanone or cycloheptanone, AlCl_3_, DCE or toluene, Δ (3–24 h, 32–87%) or MWI (10 min, 70–83%); (*iv*) dimedone, piperidine, EtOH, rt, 12 h, 69–76%; (*v*) (a) SOCl_2_, MWI, 30 min, 88%; (b) hydroxybenzaldehyde derivative, pyridine, DCM, rt, 12 h, 85–93%; (*vi*) dimedone, K_2_CO_3_, EtOH, MWI, 6 min, 85–89%.

Other tri- and tetracylcic pyranotacrines with a diverse anti-AD activity have been studied (Scheme 4A) [71,77,78,79]. The 4*H*-pyran core was formed by either a one-step reaction of ethyl benzoylacetate with benzaldehyde derivative and malononitrile in the presence of piperidine (in the case of **17a**–**p** [77]) or a two-step sequence in which the appropriate imidazole- or quinolinecarboxaldehyde was first condensed with malononitrile and then treated with a 1,3-dicarbonyl (in the case of **17q**,**t**,**u**,**x**–**ab,** and **18a**,**c**) in the presence of piperidine (Scheme 4B,C) [71,78]. Alternatively, for the sulfamoyl-containing pyranotacrines **16f**–**t**, SOCl_2_ and microwave irradiation was first used to make *p*-sulfamoylbenzoyl chloride from the corresponding acid. Esterificaton with hydroxybenzaldehydes then gave sulfamoylbenzoate benzaldehydes, which were subjected to a three-component Aldol-Michael-cyclization sequence with malononitrile and dimedone under microwave irradiation to give intermediates **18f**–**j** (Scheme 4D) [79]. In all cases, a final Friedländer reaction with the appropriate cycloalkanone in the presence of AlCl_3_ gave the target compounds **15a**–**ad** and **16a**–**t** as racemic mixtures (Scheme 4B–D).

Eghtedari et al. found that all compounds **15a**–**p** showed IC_50_ values (*Ee*AChE) < 6 µM. The best inhibitor **15i**, bearing a R_1_ 3-bromophenyl substituent was 5-fold more potent than tacrine with an IC_50_ = 69 nM and showed a mixed-type inhibition based on the Lineweaver-Burk plot. SAR suggested that the best inhibition was achieved with a R_1_ 2- or 3-bromo/chlorophenyl substituent. Molecular modeling predicted **15i** to bind to the CAS of AChE near the catalytic triad. The predominant binding interaction was predicted to be the hydrophobic/π-π interactions, but an H-bond between the amino group and His440 was also noted. While **15i** was predicted to cross the BBB (0.80 probability by the online admetSAR server), it was only weakly neuroprotective against H_2_O_2_-induced injury in PC12 cells and was moderately toxic to HepG2 cells (but still less toxic than tacrine) [77]. All synthesized sulfamoyl-containing pyranotacrines **16f**–**t** were potent *Ee*AChEi (IC_50_ < 150 nM), but **16l** was the most potent (IC_50_ = 10 nM) and was 5.5-fold more potent than tacrine (IC_50_ = 55 nM) (Table 2). In general, the cyclohexyl moiety was favored over the cyclopentyl and cycloheptyl derivatives, and the p-sulfamoylbenzoate moiety was favored at the 4-position among this series. However, no molecular modeling or X-ray crystallography was performed to visualize the interaction of the inhibitors with the enzyme. Of the 15 compounds evaluated, 10 showed some degree of antioxidant activity [79].

However, modification to the imidazo- or quinolinopyranotacrines was found to weaken the inhibition. For example, compared to tacrine (*Ee*AChE IC_50_ = 89.8 nM), all imidazopyranotacrines **15q**–**t** and **16a,b** (Table 2) were weaker inhibitors of AChE [71]. For the quinolinopyranotacrines, constructed due to the prevalence of the 2-chloroquinolin-3-yl moiety in many pharmacologically active compounds, the most potent AChEi were **15x** and **15ac** (*Ee*AChE IC_50_ = 480 and 470 nM, respectively, compared to tacrine IC_50_ = 190 nM) [78]. While lacking as AChEi, imidazo- and quinolinopyranotacrines have other beneficial properties. All of the imidazopyranotacrines showed high antioxidant capacity, with all but one compound exhibiting between 1.70–2.34 Trolox equivalents by the ORAC assay. Imidazopyranotacrine **15t**, despite a weak AChE inhibition (IC_50_ = 38.7 µM), was particularly promising given its high antioxidant capacity and low hepatotoxicity compared to tacrine in HepG2 cells (non-toxic at 1 mM) [71]. In addition, **15ac** was able to significantly inhibit *Ee*AChE-induced Aβ_1-40_ aggregation, and both **15x** and **15ac** were less hepatotoxic than tacrine in HepG2 cells, with **15x** being 27-fold less toxic. Moreover, **15x** and **15ac** showed promising neuroprotection in SH-SY5Y cells against oxidative stress, Aβ_1-40_ aggregation, and tau-phosphorylation [78].

## 6. Pyridino-, Indolo-, and Quinoxalinotacrines

**Scheme 5:**
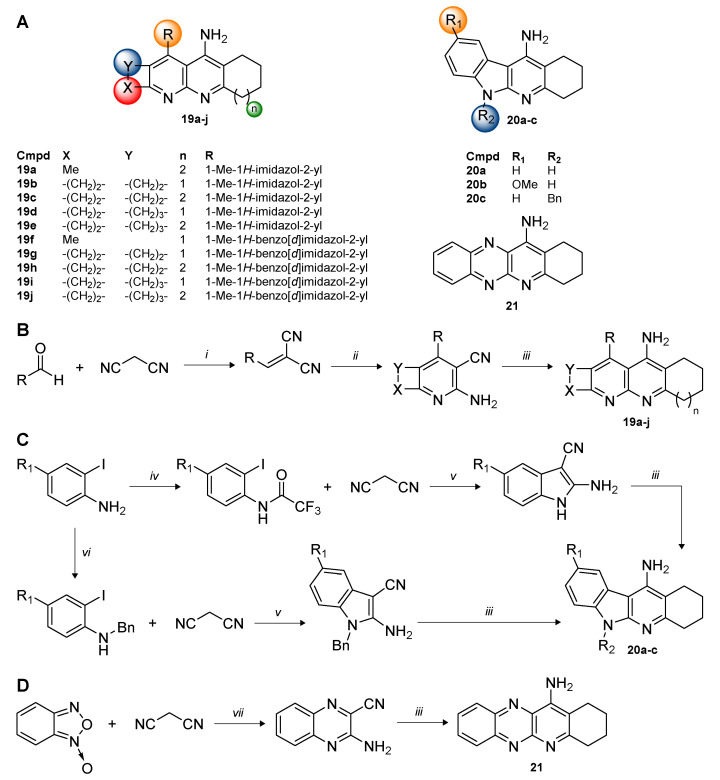
(**A**) Structure of pyridino- (**19a**–**j**), indolo- (**20a–c**), and quinoxalinotacrines **21** [80,81,82]. (**B**) Synthesis of pyridinotacrines **19a**–**j**. (**C**) Synthesis of indolotacrines **20a**–**c**. (**D**) Synthesis of quinoxalinotacrine **21**. Reagents and conditions: (*i*) Piperidine, EtOH, rt; (*ii*) acetone or cyclohexanone or cycloheptanone, NH_4_OAc, AcOH, Δ; (*iii*) cyclohexanone or cycloheptanone, AlCl_3_, DCE, Δ (18 h, 72–95%) or MWI (2 h, 16–88%); (*iv*) TFAA, TEA, THF, rt, 12 h, 97–99%; (*v*) *L*-Pro or picolinic acid, K_2_CO_3_, CuI, DMSO/H_2_O, 60 °C (12 h, 48–90%) or MWI (12 h, 26%); (*vi*) (a) benzaldehyde, MeOH, rt, 12 h, 97%; (b) NaBH_3_CN, AcOH/MeOH, rt, 12 h, 75%; (*vii*) (a) TEA, DMF, rt, 1.5 h, 75%; (b) Na_2_S_2_O_4_, H_2_O/MeOH, 50 °C, 3 h, 87% [83].

Replacement of the aromatic *A*-ring of tacrine with nitrogen heterocycles has resulted in pyridino-, indolo-, and quinoxalinotacrines (Scheme 5A) [80,81,82]. Pyridinotacrines **19a**–**j** were prepared from 1-methyl-1*H*-(benz)imidazol-2-carbaldehyde by condensation with malononitrile followed by a reaction with the appropriate enolizable ketone (acetone, cyclohexanone, or cycloheptanone) and NH_4_OAc in AcOH to yield 2-amino-3-cyanopyridine intermediates. The Friedländer reaction with the appropriate cycloalkanone in the presence of AlCl_3_ gave the target compounds (Scheme 5B) [80]. Indolotacrines **20a**–**c** were designed to fuse a 2-aminoindole-3-carbonitrile scaffold, which contains common pharmacophores of MAOi, and tacrine/7-methoxytacrine. The starting commercially available or easily made 2-iodoanilines could either be trifluoroacetylated with TFAA or benzylated with reductive amination using benzaldehyde and NaBH_3_CN. In either case, the 2-aminoindole-3-carbonitrile core was formed via Cu-catalyzed cyclization with malononitrile, which, upon the tFriedländer reaction with cyclohexanone and AlCl_3_ gave the desired compounds (Scheme 5C) [81]. Quinoxalinotacrine **21** was prepared from the Friedländer reaction between 3-amino-2-quinoxalinecarbonitrile [83] and cyclohexanone in the presence of AlCl_3_ [82].

Modification to these *N*-heterocyclic tacrine scaffolds generally comes with a reduction in AChE inhibition and mixed other properties. For example, all pyridinotacrines **19a**–**j** reported by Boulebd et al. [80] showed a weaker inhibition of *Ee*AChE than tacrine (IC_50_ = 30 nM), albeit over a fairly narrow range with IC_50_ = 310–620 nM. While not the most potent AChEi, compound **19d** (IC_50_ = 500 nM) is noteworthy in that it showed no toxicity to HepG2 cells at concentrations as high at 1 mM. The authors hypothesized that reduced toxicity compared to tacrine was due to the fully substituted pyridine ring (no C-H bonds), which blocked oxidation to the reactive iminoquinone metabolite associated with hepatotoxicity in tacrine. However, none of the synthesized compounds displayed significant antioxidant activity [80]. Indolotacrine **20b** showed that *h*AChE IC_50_ = 1.5 µM, making it more potent than 7-methoxytacrine (IC_50_ = 10 µM) but less potent than tacrine (IC_50_ = 320 nM). In addition, **20b** showed a promising inhibition of MAO-A (IC_50_ = 490 nM) and was predicted to cross the BBB (PAMPA-BBB, *P_e_* = 6.6 × 10^−6^ cm/s), but it lacked antioxidant activity and was more cytotoxic to a CHO-K1 cell line and more hepatotoxic to a HepG2 cell line than both tacrine and 7-methoxytacrine [81]. Lastly, compared to tacrine (*h*AChE IC_50_ = 374 nM), quinoxalinotacrine **21** was a significantly weaker *h*AChE inhibitor (IC_50_ = 22.0 µM). Molecular modeling predicted **21** interacted with *h*AChE in the mid-gorge region with the cyclohexyl ring oriented toward the CAS forming alkyl-π interactions with Trp86 and the phenyl and pyridinyl rings oriented towards the PAS forming π-π interactions with Tyr341 and Tyr337, respectively. Moreover, noted were key H-bonds between the amino group and pyrazine nitrogen and Tyr124 and Asp74. An initial in silico analysis predicted **21** had favorable ADME properties for CNS action (MW < 450, hydrogen-bond donors < 3, hydrogen-bond acceptors < 7, number of hydrogen bond donor < 5, Van der Waals surface area of polar nitrogen and oxygen atoms < 90, number of rotatable bonds < 8, hydrogen bonds < 8, logBB = −0.332) and that **21** (and 10 possible metabolites) showed no potential hepatotoxicity. Subsequent screening in HepG2 cells showed that **21** first showed a significant reduction in cell viability at 300 µM (for comparison, tacrine showed a significant reduction in cell viability at 30 µM). Additionally, **21** showed neuroprotection in SH-SY5Y cells against ROS (oligomycin A/rotenone-induced) and tau hyperphosphorylation (okadaic acid-induced) at concentrations as low as 0.1 µM, but the effect diminished above 1 µM [82].

## 7. Pyrrolo-, Pyrazolo-, and Furanotacrines; Pyrazolophthalazine Tacrines

**Scheme 6:**
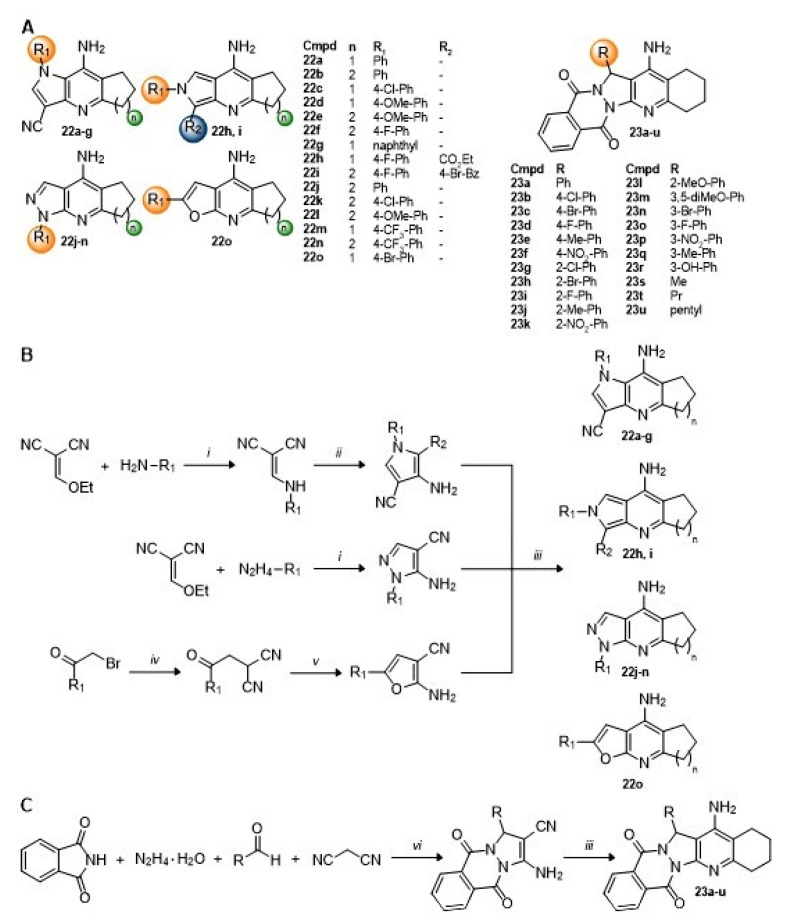
(**A**) Structure of pyrrolo- (**22a**–**i**), pyrazolo- (**22j**–**n**), and furanotacrines **22o**, as well as pyrazolophthalazine tacrines **23a**–**u** [84,85]. (**B**) Synthesis of pyrrolo- (**22a**–**i**), pyrazolo- (**22j**–**n**), and furanotacrine **22o**. (**C**) Synthesis of pyrazolophthalazine tacrines **23a**–**u**. Reagents and conditions: (*i*) EtOH, rt, 30 min, >80%; (*ii*) X-CH_2_-R_2_ (where X = Cl or Br, Y = CN, CO_2_Et, or 4-Br-Bz) TEA, Δ, 15–30 min, 63–91%; (*iii*) cyclopentanone or cyclohexanone, AlCl_3_, DCE or DCM, Δ (8–24 h, 30–95%) or MWI (30–32 min, 45–87%); (*iv*) malononitrile, 10% KOH, EtOH, rt, 30 min, 80%; (*v*) TEA, EtOH, Δ, 2 h, 67%; (*vi*) NiCl_2_·6H_2_O, EtOH, Δ, 4 h.

Again, replacement of the aromatic *A*-ring of tacrine with other nitrogen and oxygen heterocycles has resulted in pyrrolo-, pyrazolo-, and furanotacrines, as well as pyrazolophthalazine tacrines (Scheme 6A) [84,85]. The pyrrolotacrine series **22a**–**i** was constructed starting from ethoxymethylene malononitrile by a reaction with aromatic amines to form arylaminomalonitriles, which could undergo Thorpe-Ziegler cyclization to the pyrrole upon reaction with chloroacetonitrile, 4-bromophenacylbromide, or ethyl bromoacetate and TEA. A final Friedländer reaction with cycloalkanones and AlCl_3_ under microwave irradiation gave the target 2,3-fused pyrrolotacrines **22a**–**g** or 3,4-fused pyrrolotacrines **22h,i** (Scheme 6B). Likewise, the pyrazolo series was also constructed starting from ethoxymethylene malononitrile by a reaction with aromatic hydrazines to form the 5-amino-4-cyanopyrazoles followed by the Friedländer reaction with cycloalkanones and AlCl_3_ under microwave irradiation gave the target 3,4-fused pyrazolotacrines **22j**–**n** (Scheme 6B). The furanotacrine **22o** was constructed starting from malononitrile by initial alkylation with 4-bromophenacylbromide and then cyclization with TEA to the 2-amino-3-cyanofuran. A final Friedländer reaction with cyclopentanone as before gave the target compound (Scheme 6B) [84]. For **23a**–**u**, the pyrazolo[1,2-*b*]phthalazine core was constructed using a one-pot, Ni-catalyzed, four-component reaction with phthalimide, hydrazine hydrate, malononitrile, and benzaldehyde derivatives. A subsequent Friedländer reaction with cyclohexanone and AlCl_3_ gave the desired products as racemic mixtures (Scheme 6C) [85].

Compounds **22a**–**o** were shown to be potent AChEi, as all compounds presented IC_50_ values (enzyme source not specified) between 4.06–6.87 nM that are comparable to donepezil (IC_50_ = 7.23 nM). There seems to be little difference between the pyrolo-, pyrazolo-, and furanotacrines in regards to the inhibitory activity, and no additional biological properties were examined [84]. A more in depth analysis was performed on pyrazolophthalazine tacrines **23a**–**u**. Of the compounds synthesized, five showed very potent inhibition (*Ee*AChE IC_50_ < 100 nM) with **23o** (IC_50_ = 23 nM), bearing a *m*-fluorophenyl substituent, and **23l** (IC_50_ = 49 nM), bearing an *o*-methoxyphenyl substituent, being the most potent and being 16-fold and 7.5-fold more potent than tacrine, respectively (Table 3). SAR showed that the phenyl substituted compounds outperformed the alkyl substituted ones, and *o*/*m*/*p*-substituted phenyl substituents outperformed the non-substituted phenyl counterpart. The Lineweaver-Burk plot showed that **23l** as a mixed-type inhibitor, and theoretical calculations predicted it to be CNS active (0.9260 probability by the online admetSAR server). Molecular modeling showed the *R*-enantiomer of **23l** to interact more favorably with *Tc*AChE, specifically at the PAS via the π-π stacking with Tyr120 and Trp278 and H-bonding with Tyr69. Additionally, **23l** showed that the moderate ability to inhibit both self- and AChE-induced Aβ aggregation, was less toxic than tacrine with HepG2 cell viability remaining high (83%) at 300 µM, and exhibited a slight antioxidant activity as it protected PC12 cells from H_2_O_2_-induced death at 100 µM [85].

**Scheme 7:**
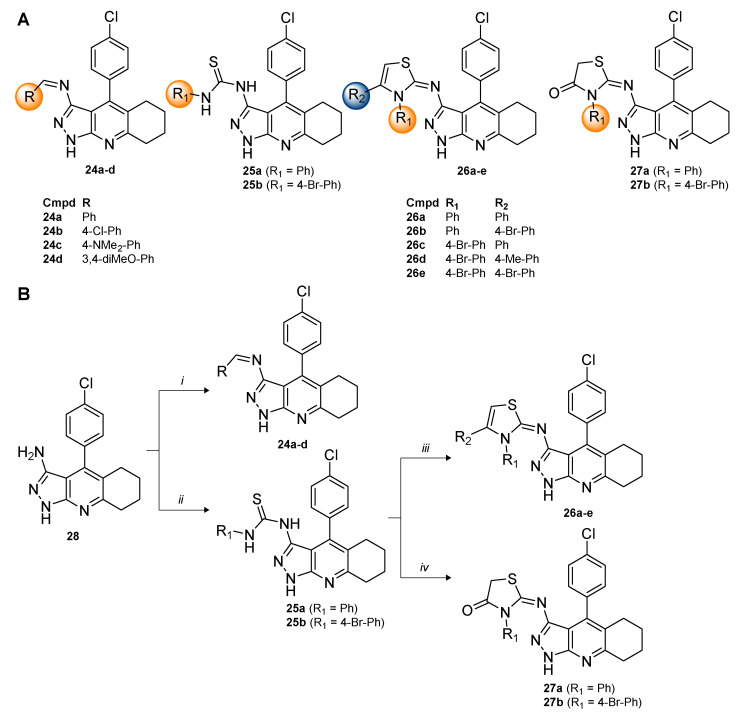
(**A**) Structure of pyrazolotacrines **24a**–**d**, **25a,b**, **26a**–**e,** and **27a,b** [86]. (**B**) Synthesis of pyrazolotacrines **24a**–**d**, **25a,b**, **26a**–**e,** and **27a,b**. Reagents and conditions: (*i*) R-CHO, piperidine, EtOH, Δ, 4 h, 80–90%; (*ii*) R_1_-NCS, EtOH, Δ, 4–6 h, 83–89%; (*iii*) R_2_-COCH_2_Br, NaOAc, 1,4-dioxane, Δ, 4–6 h, 60–92%; (*iv*) ethyl bromoacetate, NaOAc, 1,4-dioxane, Δ, 4–6 h, 68–79%.

**Figure 6:**
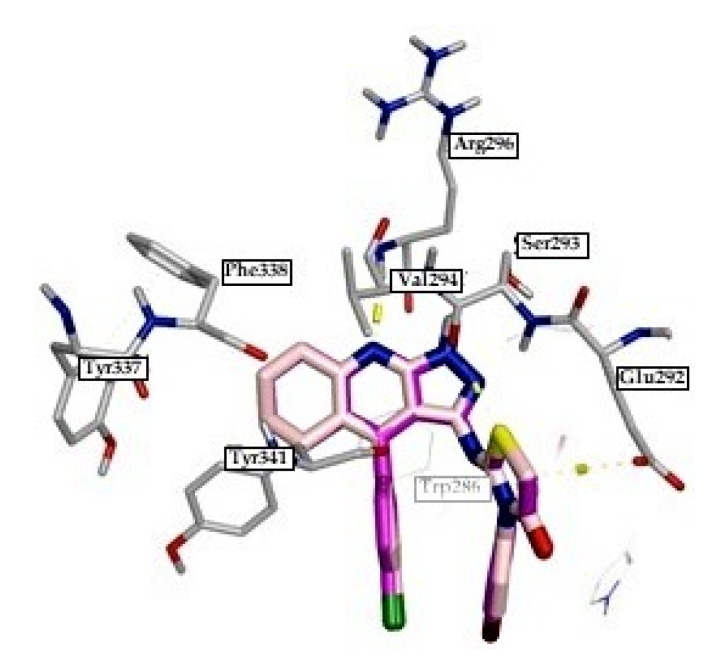
Molecular modeling of **27a** (rose) and **27b** (purple) with *h*AChE showing the interaction with the PAS (adapted from [86], with permission from Elsevier). Key interactions noted are the tricyclic core showing favorable hydrophobic interactions with Tyr341, Tyr337, Phe338, and Val294, the 4-chlorophenyl substituent showing hydrophobic interactions with Tyr341 and Trp286, the quinolinyl nitrogen showing an H-bond with Arg296, and the thiazolidinone moiety showing favorable interactions with Glu292 and Ser293.

An additional series of pyrazolotacrines **24a**–**d**, **25a,b**, **26a**–**e**, and **27a,b** have been very recently reported (Scheme 7A) [86]. The design rationale for these compounds was to merge the 4-chlorophenyltetrahydroquinoline moiety for AChE inhibition with the pyrazole and thiourea moieties of known COX-2 inhibitors (e.g., celecoxib) to potentially modulate the ACh and ROS/inflammatory aspects of AD. Starting from previously prepared amino pyrazolotacrine intermediate **28** [87], condensation with benzaldehyde derivatives or phenyl isothiocyanates gave the imines **24a**–**d** and thioureas **25a,b**. A further reaction of **25a,b** with phenacyl bromides or ethyl bromoacetate afforded the thiazolidines **26a**–**e** and thiazolidinones **27a,b**, respectively (Scheme 7B) [86]. Interestingly, the authors chose to assess in vitro AChE inhibition through a percentage increase in contraction of frog’s Rectus abdominis, as opposed to the more common Ellman assay [60], making a direct comparison to other compounds described in this review difficult. Suffice it to say, all compounds showed AChE inhibitory activity that was comparable or better than tacrine except **25a**. In particular, **24b**, **26e**, and **27a,b** were all roughly at least twice as active as tacrine. COX-2 inhibition assays indicated that **24b**, **26e**, and **27a,b** showed IC_50_ values between 0.76–0.89 µM, which was comparable to celecoxib (IC_50_ = 0.84 µM). Of note, **26e** showed remarkable selectivity towards COX-2 over COX-1 (13-fold more potent towards COX-2, better than celecoxib), which is beneficial for reducing adverse renal and gastrointestinal side effects [88]. The hepatoxicity was investigated through determination of serum glutamic pyruvic transaminase (SGPT) levels, and all compounds proved less hepatotoxic than tacrine. Molecular modeling with *h*AChE showed similar interactions for all compounds. For example, **27a,b** (Figure 6) was predicted to interact in the PAS with the tricyclic core showing favorable hydrophobic interactions with Tyr341, Tyr337, Phe338, and Val294. The 4-chlorophenyl substituent also showed hydrophobic interactions with Tyr341 and Trp286, and the quinolinyl nitrogen was predicted to H-bond with Arg296. The thiazolidinone moiety showed a favorable interaction with Glu292 and Ser293 [86].

## 8. Urea and Thiourea Tacrines

**Scheme 8:**
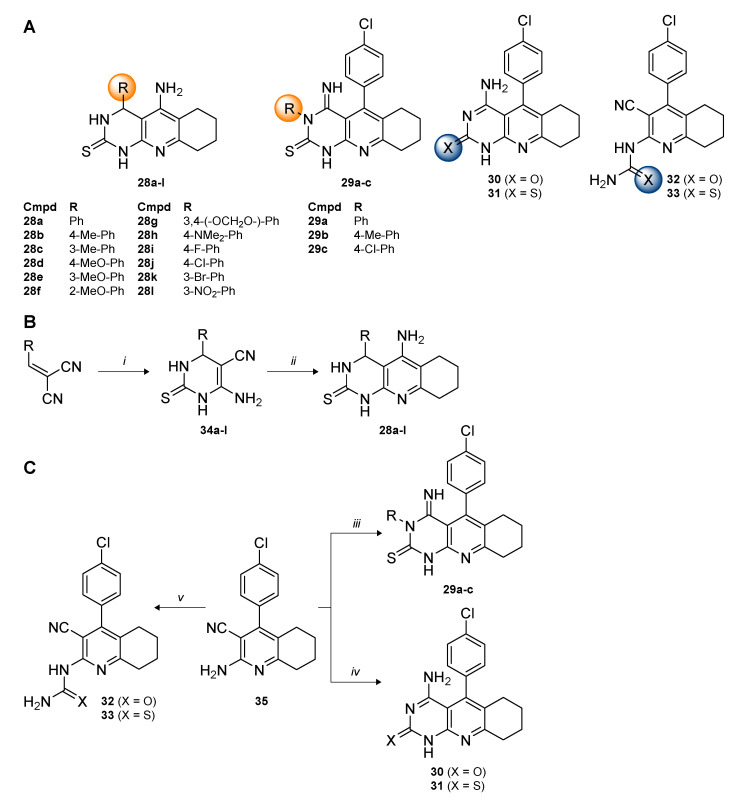
(**A**) Structure of thiourea tacrines **28a**–**l**, **29a**–**c**, **31**, and **33** and urea tacrines **30** and **32** [89,90]. (**B**) Synthesis of thiourea tacrines **28a**–**l**. (**C**) Synthesis of thiourea and urea tacrines **29a**–**c** and **30**–**33**. Reagents and conditions: (*i*) Thiourea, NaOMe, EtOH, Δ, 12 h, 10–29%; (*ii*) cyclohexanone, AlCl_3_, DCE, MWI, 1–2 h, 46–70%; (*iii*) SCN-R, pyridine, Δ, 10 h, 85–86%; (*iv*) urea or thiourea, 300 °C, 1 h, 64–74%; (*v*) urea or thiourea, 200 °C, 1 h, 76–83%.

Still further replacement of the aromatic *A*-ring of tacrine with additional heterocycles has resulted in thiourea and urea tacrines (Scheme 8A) [89,90]. Thiourea tacrines **28a**–**l** were designed to combine tacrine and 3,4-dihydropyrimidin-2(1*H*)-thiones, known as calcium channel blockers [91,92] that have also shown metal-mediated, Aβ-related neuroprotection [93], into a multifunctional scaffold. The 3,4-dihydropyrimidin-2(1*H*)-thione moiety **34a**–**l** was prepared by the reaction of substituted arylidenemalononitriles with thiourea in the presence of NaOMe. The exocyclic nitrile and amine then readily underwent the Friedländer reaction with cyclohexanone in the presence of AlCl_3_ to afford the target compounds as racemic mixtures (Scheme 8B) [89]. The synthesis of **29a**–**c** and **30**–**33** was done in one-step from the 2-amino-3-cyanotetrahydroquinoline **35**. Precursor **35** could be condensed with aryl isothiocyanates to afford cyclized thiourea tacrines **29a**–**c**, or it could be fused with urea or thiourea under high temperature (300 °C) to afford cyclized urea and thiourea tacrines **30** and **31** or moderate temperature (200 °C) to afford the open chain urea and thiourea tacrines **32** and **33** (Scheme 8C) [90].

Thiourea tacrines **28a**–**l** showed a promising biological profile. The most potent *h*AChE inhibitor was **28k** (IC_50_ = 37.3 nM), which had a 3-bromophenyl substituent and was 10-fold more potent than tacrine (IC_50_ = 374 nM). In addition, **28e**, which had a 3-methoxyphenyl substituent, was the next most potent but significantly weaker (IC_50_ = 3.05 µM). All other compounds in this series were significantly weaker AChEi (IC_50_ > 5 µM). Both **28e** and **28k** showed non-competitive inhibition by Lineweaver-Burk plot analysis, suggesting PAS interaction. This was confirmed by molecular modeling with *h*AChE where (*R*)-**28k** was predicted to bind to the PAS by π-π interactions with the phenyl ring and Trp286 and Tyr72 and the aminopyridine ring and Tyr341. H-bonds were also seen between the tacrine-like amine and Asp74, and the bromo substituent was involved in halogen bonds. Overall binding of the *S*-enantiomer was similar. Notably, **28e** was also predicted to bind to the PAS, but it lacked any binding contribution from the methoxy substituent, which may explain its reduced activity. In addition, compared to known calcium channel blocker nimodipine (49.62%), both **28e** and **28k** showed similar inhibition of Ca^2+^ influx (30.40% and 42.23%), but neither **28e** nor **28k** showed significant inhibition of Aβ_1-42_ self-induced aggregation. In addition, **28e** was particularly promising in that it displayed no hepatotoxicity to HepG2 cells at concentrations up to 300 µM [89]. For **29a**–**c** and **30**–**33**, Ragab et al. chose to assess the in vitro AChE inhibition again through the less common percentage increase in contraction of frog’s Rectus abdominis, making direct comparison to other compounds described in this review difficult. However, it is still noted that all compounds showed promising AChE inhibitory activity except compound **29b**. Hepatoxicity was investigated through the determination of SGPT levels and glutathione (GSH) levels, and all compounds proved less hepatotoxic than tacrine. In silico calculations predicted that all compounds had promising drug-like characteristics [90].

## 9. Amido-, Amino-, and Iminotacrines

**Scheme 9:**
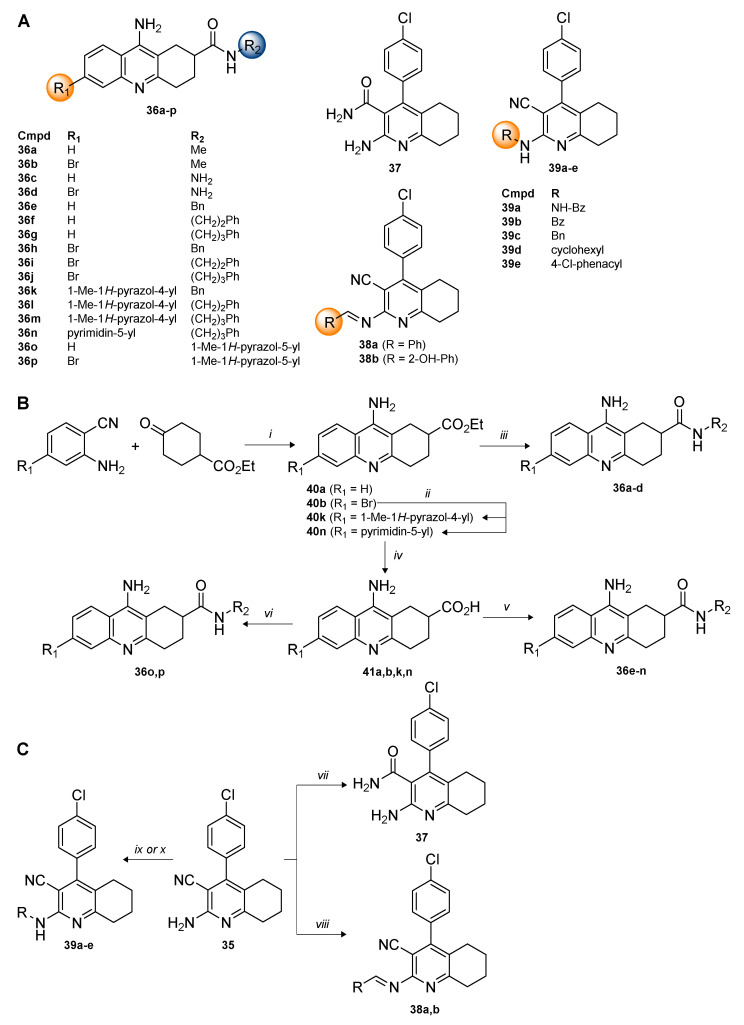
(**A**) Structure of amido- (**36a**–**p**), amino- (**37** and **39a**–**e**), and iminotacrines **38a,b** [90,94]. (**B**) Synthesis of amidotacrines **36a**–**p**. (**C**) Synthesis of amino- (**37** and **39a**–**e**) and iminotacrines **38a,b**. Reagents and conditions: (*i*) BF_3_·Et_2_O, toluene, Δ, 4 h, 84%; (*ii*) 1-methylpyrazole-4-boronic acid pinacol ester or 5-pyrimidineboronic acid pinacol ester, Na_2_CO_3_, Pd(PPh_3_)_4_, 1,4-dioxane/H_2_O, Δ, 2 h, 52%; (*iii*) NH_4_OH or N_2_H_4_·H_2_O, MeOH, 60 °C, 2–3 h, 73–85%; (*iv*) LiOH·H_2_O, THF/H_2_O/MeOH, rt, 3 h, 79–90%; (*v*) benzylamine or phenylethylamine or phenylpropylamine, T_3_P, TEA, DMF, 60 °C, 4 h, 59–67%; (*vi*) 5-amino-1-methyl-1*H*-pyrazole, HATU, DIPEA, DMF, rt, 12 h, 63–71%; (*vii*) 70% H_2_SO_4_, Δ, 5 h; (*viii*) R-C_6_H_4_-CHO, pyridine, Δ, 6 h, 87–90%; (*ix*) NH_2_NH-Bz, Δ, 1 h, 84%; (*x*) X-R (where X = Cl or Br), 1,4-dioxane, Δ, 5–10 h, 69–89%.

Amidotacrines **36a**–**p** were designed to explore the effect of adding an amide moiety to the 2-position of the cyclohexyl *C*-ring of tacrine, an underexplored SAR [94]. Meanwhile, amino- (**37** and **39a**–**e**) and iminotacrines **38a,b** focus on replacing the aromatic *A*-ring of tacrine with amine or imine moieties (Scheme 9A) [90]. Synthetically, amidotacrines were accessed via the key intermediate ethyl 2-tacrine carboxylate (**40a**) or the corresponding 6-bromo analog **40b**, which were prepared by the Friedländer reaction with 2-aminobenzonitriles, ethyl 4-oxocyclohexanecarboxylate, and BF_3_·Et_2_O (Scheme 9B). To investigate the effect of different heterocyclic substituents at the 6-position, **40b** was subjected to Pd-catalyzed Suzuki-Miyaura cross-coupling with the corresponding pyrazolo- or pyrimido-boronic ester to give **40k** and **40n**, respectively. Nucleophilic acyl substitution of **40a**/**b** with methylamine or hydrazine afforded **36a**–**d**. Alternatively, **40a,b,k,n** could be saponified to acids **41a,b,k,n** and coupled to various phenyalkylamines using T_3_P to afford **36e**–**n** or coupled to 5-amino-1-methyl-1*H*-pyrazole using HATU to afford **36o,p** [94]. Compound **35**, for the synthesis of amino- and iminotacrines, could be hydrolyzed with 70% sulfuric acid to afford **37**, condensed with aryl aldehydes or benzoic acid hydrazide to afford Schiff bases **38a,b** or benzoylhydrazide **39a**, and acylated with benzoyl chloride or alkylated with different alkyl halides to afford derivatives **39b**–**e** (Scheme 9C) [90].

**Figure 7:**
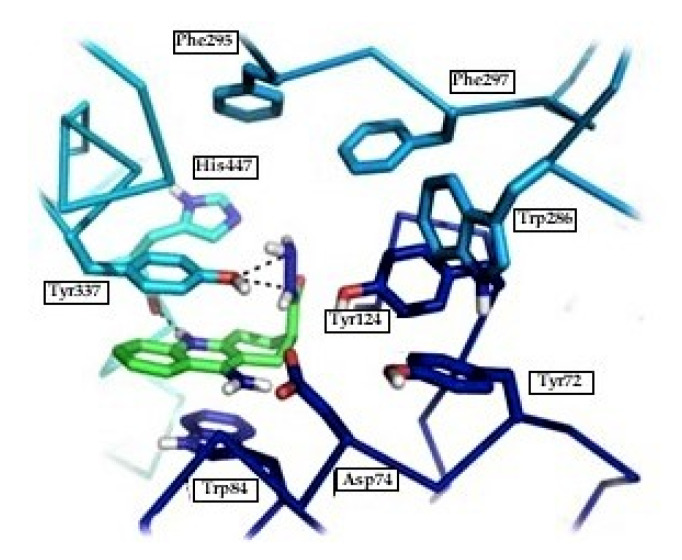
Molecular modeling of **36c** with *Tc*AChE showing the interaction with the CAS (adapted from [94], with permission from Elsevier). Key interactions noted are the tacrine moiety π-π stacking with Trp84 and Tyr337 and H-bonding to His447 and the hydrazide H-bonding with Tyr337.

With two exceptions, all amidotacrines **36a**–**p** were more potent than tacrine (*Ee*AChE IC_50_ = 94.69 nM), and the best inhibitor was **36g** (IC_50_ = 5.17 nM), which had no substituent at the 6-position of the tacrine core and a phenylpropylamide at the 2-position (Table 4). SAR indicated that the phenylpropylamide at the 2-position gave the best inhibition of AChE, the bromo substituent at the 6-position seemed to have little effect, and a heterocycle (pyrazolo or pyrimido) at the 6-position decreased inhibition. Of note, **36c** with no substituent at the 6-position and the hydrazide at the 2-position was a potent inhibitor (IC_50_ = 12.97 nM). Molecular modeling with *Tc*AChE showed that most compounds investigated positioned the tacrine moiety in the CAS, exhibiting π-π stacking with Trp84 and Tyr337 and H-bonding to His447, and oriented the 2-position towards the PAS allowing the amides to make key contacts with residues there. Of note, **36g** was positioned to allow the amide nitrogen to H-bond with Tyr124, and **36c** was positioned to allow the hydrazide to H-bond with Tyr337 (Figure 7). Compound **36c** was particularly promising as it uniquely, among the compounds tested, showed no cytotoxicity to HEK-293 cells or HepG2 cells at concentrations up to 300 µM [94]. Amino- and iminotacrines **37**, **38a,b**, **39a**–**e**, also prepared by Ragab et al. and tested in an uncommon AChEi assay (see above), showed promising AChE inhibitory activity with the exception of compounds **39a,b**. Compound **39c**, with a 2-benzylamino substituent, had the highest activity (nearly 2-fold more active than tacrine). Molecular modeling of **39c** with *h*AChE showed H-bonding between the benzylamino NH and Asp93 and between the nitrogen of the CN group and Tyr96, as well as hydrophobic interactions with various amino acids. This compound also was less hepatotoxic than tacrine and predicted to have promising drug-like characteristics [90].

## 10. Naphthalene and Naphthoquinone Pyranopyrimidinones/Pyrimidinimines

**Scheme 10:**
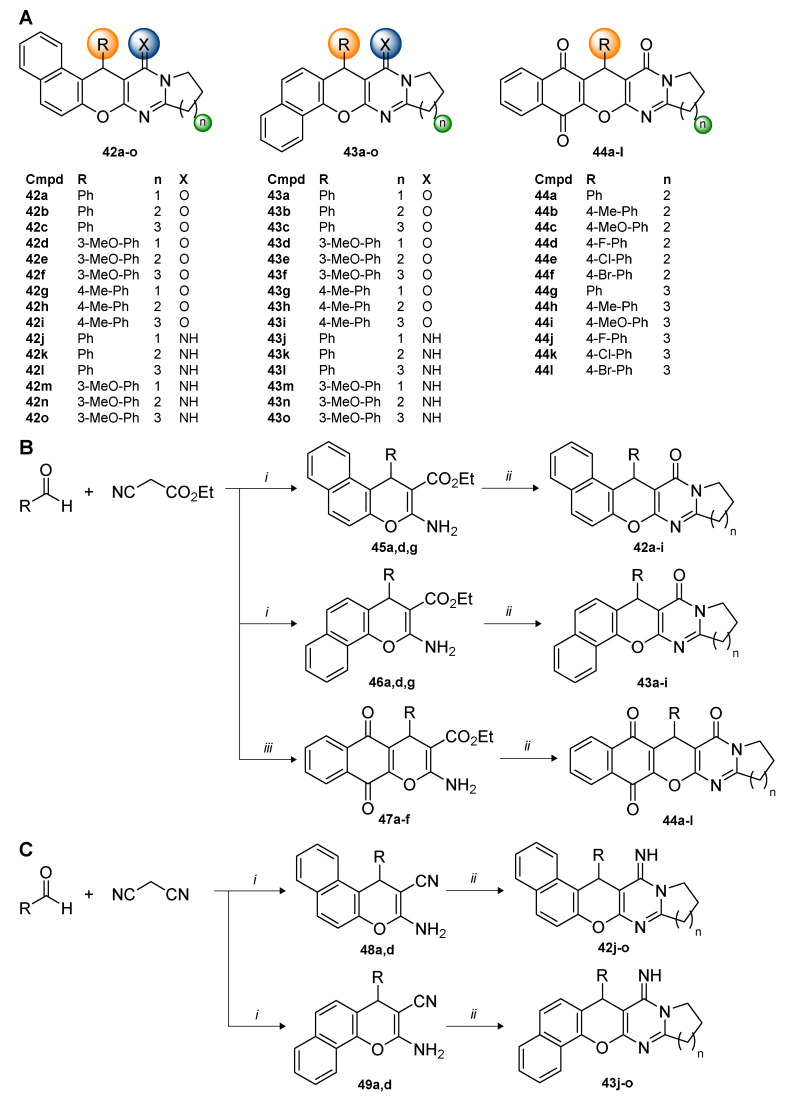
(**A**) Structure of naphthalene pyranopyrimidinones **42a**–**i** and **43a**–**i**, naphthoquinone pyranopyrimidinones **44a**–**l**, and naphthalene pyrimidinimines **42j**–**o** and **43j**–**o** [95,96,97]. (**B**) Synthesis of naphthalene pyranopyrimidinones **42a**–**i** and **43a**–**i** and naphthoquinone pyranopyrimidinones **44a**–**l**. (**C**) Synthesis of naphthalene pyranopyrimidinimines **42j**–**o** and **43j**–**o**. Reagents and conditions: (*i*) 2-Naphthol or 1-naphthol, piperidine, EtOH, MWI, 10 min, 68–95%; (*ii*) γ-butyrolactam or δ-valerolactam or ε-caprolactam, POCl_3_, DCE or PhBr, MWI, 15 min, 70–96%; (*iii*) potassium phthalimide-*N*-oxyl, H_2_O, Δ, 30–60 min, >90%.

Pyranopyrimidinones and pyranopyrimidinimines **42a**–**o**, **43a**–**o**, and **44a**–**l** (Scheme 10A) focus on two key modifications to the tacrine scaffold to modulate AChE inhibition and antioxidant activity: (1) Replacement of the aromatic *A*-ring with a fused pyranonapthalene or pyranonaphthoquinone moiety and (2) change of the central aromatic *B*-ring from an aminopyridine to a pyrimidinone or pyrimidinimine [95,96,97]. Several examples of the former modification have been presented herein, but the latter remains underexplored. The transition to a pyrimidinone or pyrimidinimine is based on the fact that quinazolinone and quinazolinimine derivatives inspired by naturally occurring alkaloids structurally resemble tacrine (e.g., deoxyvasicinone) and have shown promising activity as AChEi [98,99,100,101,102]. The naphthalene pyranopyrimidinones **42a**–**i** and **43a**–**i** were prepared using a three-component, one-pot reaction with ethyl cyanoacetate, benzaldehydes, and 2- or 1-naphthol in the presence of piperidine to give **45a,d,g** and **46a,d,g**, followed by condensation with lactams in the presence of POCl_3_ (Scheme 10B) [95]. Similarly, the pyranopyrimidinimies **42j**–**o** and **43j**–**o** were prepared using the same three-component, one-pot reaction with the exception of malononitrile in place of ethyl cyanoacetate to give **48a,d** and **49a,d**. Subsequent condensation with lactams in the presence of POCl_3_ gave the desired compounds (Scheme 10C) [96]. Moreover, in a similar fashion, naphthoquinone pyranopyrimidinones **44a**–**l** were prepared using a three-component, one-pot reaction with ethyl cyanoacetate, benzaldehydes, and 2-hydroxynaphthalene-1,4-dione in the presence of potassium phthalimide-*N*-oxyl [103] to give **47a**–**f**. Condensation with lactams in the presence of POCl_3_ as before gave the desired compounds (Scheme 10B) [97].

Among these three related series of compounds, a strong inhibition of AChE, such as tacrine, was generally maintained. For example, **42e**, containing a piperidine-fused ring, 3-methoxyphenyl substituent, and derived from 2-naphthol was the most potent of the naphthalene pyranopyrimidinones regarding *Ee*AChE inhibition (IC_50_ = 30.5 nM) and was comparable to tacrine (IC_50_ = 44.3 nM) (Table 5) [95]. In addition, **42n**, also containing a piperidine-fused ring, 3-methoxyphenyl substituent, and derived from 2-naphthol, was the most potent of the pyranopyrimidinimines (IC_50_ = 3.2 nM), and this was followed closely by **42o** (IC_50_ = 5.3 nM) bearing an azepane-fused ring (Table 5) [96]. Several of the naphthoquinone pyranopyrimidinones were similar in potency to tacrine (*Ee*AChE IC_50_ = 31 nM). Of interest were the most potent **44f** (IC_50_ = 44 nM), which contained a piperidine-fused ring and 4-bromophenyl substituent, and the similarly potent **44a** (IC_50_ = 52 nM), which contained a piperidine-fused ring and phenyl substituent [97]. Notably, across all three series, even the worst inhibitor was still fairly potent (IC_50_ ≈ 250–550 nM).

**Figure 8:**
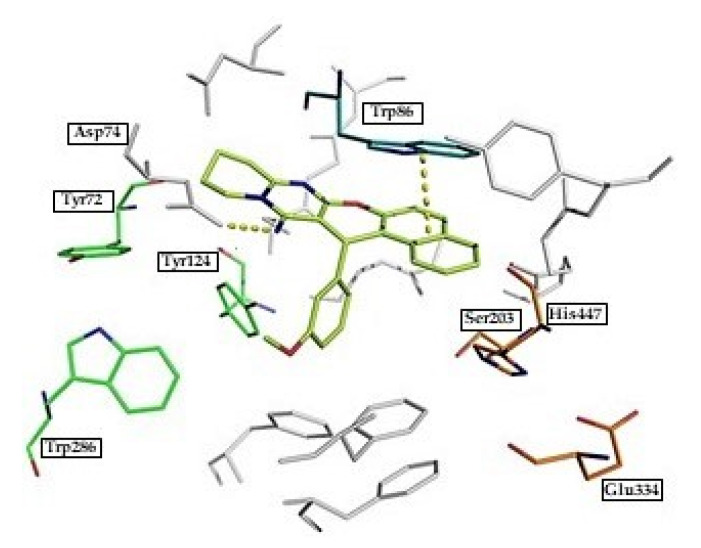
Molecular modeling of (*S*)-**42n** with *h*AChE showing the interaction with the mid-gorge region (adapted from [96], © 2016 WILEY-VCH Verlag GmbH & Co. KGaA, Weinheim). Key interactions noted are the π-π stacking between Trp86 and the benzochromeno moiety and an H-bond between Asp74 and the imino moiety.

Compound **42e** displayed a mixed-type inhibition by the Lineweaver-Burk plot analysis. Additionally, it displayed potent antioxidant activity by the ORAC assay (4.7 Trolox equivalents) and showed no toxicity in HepG2 cells at concentrations up to 1 mM, but its BBB permeability is questionable (PAMPA-BBB, *P_e_* = 3.6 × 10^−6^ cm/s) [95]. For the pyranopyrimidinimines, SAR clearly showed the 3-methoxyphenyl substituent to be more potent than the phenyl, and the 2-naphthol derived compounds to be more potent than those derived from 1-naphthol. Compound **42n** displayed noncompetitive inhibition by the Lineweaver-Burk plot analysis, which agrees with molecular modeling predicting binding of the *S*-enantiomer (but not *R*) of **42n** with the mid-gorge region of *h*AChE (Figure 8). Key interactions include a π-π stacking between Trp86 and the benzochromeno moiety and an H-bond between Asp74 and the imino moiety. Additionally, **42n** and **42o** displayed potent antioxidant activity by the ORAC assay (3.4 and 3.6 Trolox equivalents, respectively), but only **42o** showed a significant Aβ_1-42_ self-aggregation inhibition at 10 µM (40.3%) [96]. All naphthoquinone pyranopyrimidinones displayed some degree of antioxidant activity by the ORAC assay (**44a** = 2.78 Trolox equivalents), but most displayed significant toxicity to HepG2 cells. In addition, **44a** was the exception as it maintained 84.5% HepG2 cell viability at 300 µM and was 2-fold less toxic than tacrine at the same concentration [97].

## 11. Other Tacrines

**Scheme 11:**
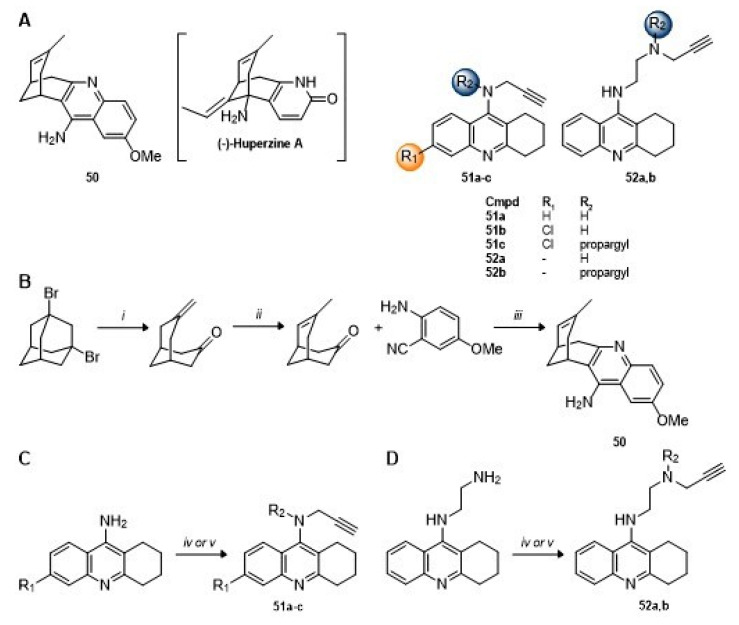
(**A**) Structure of 2-methoxyhuprine (**50**) ((−)-huperine A shown for reference) and propargyl tacrines **51a**–**c** and propargyl ethylenediamine tacrines **52a,b** [106,107]. (**B**) Synthesis of 2-methoxyhuprine (**50**). (**C**) Synthesis of propargyl tacrines **51a**–**c**. (**D**) Synthesis of propargyl ethylenediamine tacrines **52a,b**. Reagents and conditions: (*i*) 1 M NaOH, 1,4-dioxane, Δ, 18 h, 82%; (*ii*) 5% Pd/C, H_2_, EtOH, rt, 2 h, quant.; (*iii*) AlCl_3_, DCE, MWI, 2 h, 80%; (*iv*) propargyl bromide (1.1 eq), KOH or K_2_CO_3_, MeCN, rt, 12–36 h, 35–82%; (*v*) propargyl bromide (2.1 eq), KOH or K_2_CO_3_, MeCN, rt, 12–36 h, 35–82%.

This final section focuses on tacrine-based molecules with little structural similarity to those of previous sections. Huprines combine the aminoquinoline moiety of tacrine and the carbocyclic bridged moiety of huperine A (Scheme 11A), a naturally occurring sesquiterpene alkaloid AChEi. Of particular interest is huprine Y that has shown dramatic improvement compared to parent structures regarding AChE inhibition, while also showing neuroprotection against 3-nitropropionic acid-induced neurodegeneration in mice [104,105]. In constructing 2-methoxyhuprine (**50**) (Scheme 11A), Mezeiova et al. sought to maintain the beneficial properties of huprine Y while adding the reduced toxicity of 7-methoxytacrine by combining the carbocyclic bridged moiety and methoxyaminoquinoline moiety [106]. Synthetically, 1,3-dibromoadamantane was first fragmented to the ketone by reaction with NaOH at high temperature. Treatment with 5% Pd/C and H_2_ in EtOH then led to isomerization of the carbon-carbon double bond. Finally, the Friedländer reaction with 2-amino-5-methoxybenzonitrile and AlCl_3_ afforded racemic **50**, which was converted to the hydrochloride salt for biochemical testing (Scheme 11B). Regarding *h*AChE inhibition, 2-methoxyhuprine (**50**) showed a mixed-type inhibition, as determined by the Lineweaver-Burk analysis, with an IC_50_ = 2.63 µM and was more nearly 4-fold more potent than the parent 7-methoxytacrine (IC_50_ = 10 µM). However, it was 8-fold less potent than tacrine (IC_50_ = 320 nM) and 1600-fold less potent than the parent huprine Y (IC_50_ = 1.64 nM). Interestingly, molecular modeling with *h*AChE predicted the more active enantiomer to be the *S*,*S*-enantiomer. In vitro assays predicted favorable CNS permeability (PAMPA-BBB, *P_e_* = 7.64 × 10^−6^ cm/s) for **50**, but unfavorable toxicity, as in HepG2, ACHN, and SH-SY5Y cell lines, it showed significantly more toxicity than tacrine and 7-methoxytacrine (comparable to huprine Y) [106].

Propargyl tacrines **51a**–**c** and propargyl ethylenediamine tacrines **52a,b** (Scheme 11A,C,D) were prepared by mono- or dialkylation of tacrine, 6-chlorotacrine, or ethylenediamine tacrine using propargyl bromide (1.1 or 2.1 eq.) [107]. A single propargyl group attached to tacrine or 6-chlorotacirne was found to increase *Ee*AChE inhibition, as both **51a,b** were 2-fold more potent than the parent molecules (IC_50_ = 51.3 nM for **51a** vs. 104.8 nM for tacrine; 11.2 nM for **51b** vs. 23.5 nM for 6-chlorotacrine). Dipropargylation of tacrine or 6-chlorotacrine or mono- or dipropargylation of ethylenediamine tacrine was found to decrease inhibition. The Lineweaver-Burk plot indicated a mixed-type inhibition for **51a**, and both **51a,b** showed decreased hepatotoxicity compared to tacrine [107].

**Scheme 12:**
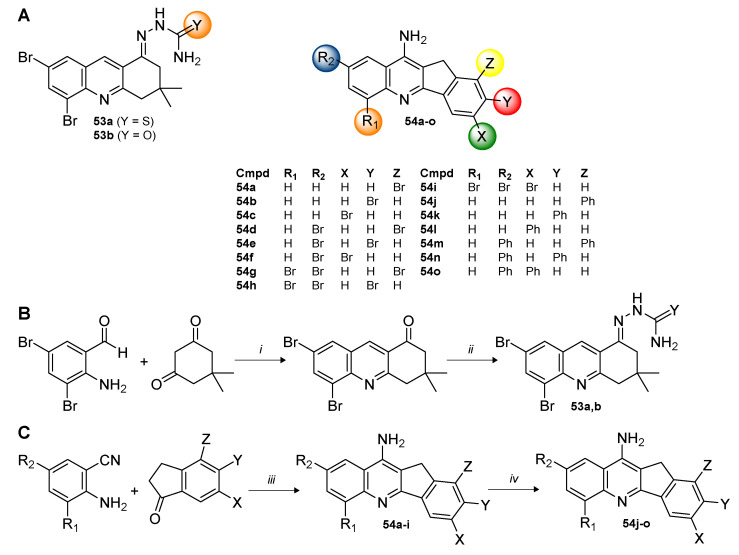
(**A**) Structure of semicarbazone tacrines **53a,b** and indenoquinolines **54a**–**o** [108,111]. (**B**) Synthesis of semicarbazone tacrines **53a,b**. (**C**) Synthesis of indenoquinolines **54a**–**o**. Reagents and conditions: (*i*) 1,4-Doxane, Δ, 24 h, 80%; (*ii*) thiosemicarbazide hydrochloride or semicarbazide hydrochloride, EtOH, Δ, 3 h, 75–79%; (*iii*) (a) InCl_3_, toluene, Δ, 24 h; (b) 2 M NaOH, Δ, 24 h, 13–50%; (*iv*) PhB(OH)_2_ (1.3 or 2.6 eq.), Pd(PPh_3_)_4_, K_2_CO_3_, 1,4-dioxane, Δ, 4 h, 80–96%.

Semicarbazone tacrines **53a,b** (Scheme 12A) were designed to incorporate semicarbazone or thiosemicarbazone at the 1-position of the tacrine tricyclic core [108]. These functional groups have diverse biological activity, and they have shown promise for AD as AChEi, metal chelators, and anti-Aβ compounds [109,110]. Condensation of 2-amino-3,5-dibromobenzaldehyde with dimedone afforded the tricyclic core, which readily condensed with thiosemicarbazide or semicarbazide in a second step to yield the target compounds **53a,b** (Scheme 12B). Inhibition studies showed that both were weak, mixed-type inhibitors of *Ee*AChE (IC_50_ = 10.30 and 8.66 µM, respectively) and were at least 200-fold less potent than tacrine (IC_50_ = 41.41 nM). Molecular modeling did predict a simultaneous CAS and PAS interaction with *Ee*AChE, and in silico calculations also suggested favorable drug-like properties and lower toxicity than tacrine [108].

Lastly, Ekiz et al. prepared indenoquinoline tacrine analogs **54a**–**o** (Scheme 12A) for evaluation as AChEi and CAi [111]. InCl_3_-catalyzed Friedländer reaction of 2-aminobenzonitrile or brominated derivatives with bromoindanones gave **54a**–**i**. These were tested directly, or further derivatized to the mono- or diphenyl compounds **54j**–**o** by Pd(PPh_3_)_4_-catalyzed Suzuki Coupling with phenylboronic acid (1.3 or 2.6 eq.) in the presence of aq. K_2_CO_3_ in dioxane (Scheme 12C). The monophenylindenoquinolines showed the most potent inhibition of *Ee*AChE (IC_50_ = 37–57 nM), which was comparable to tacrine (IC_50_ = 59 nM), indicating that phenyl substituents at the 1-, 2-, or 3-positions were favorable (Table 6). The most potent was **54j** bearing a 1-phenyl substituent (IC_50_ = 37 nM). Additional SAR analysis showed that AChE inhibition was greatly reduced in the diphenylindenoquinolines and the mono-, di-, and tribromoindenoquinolines (IC_50_ > 1 µM). The lone exception was the 1,8-dibromo compound **54d** (IC_50_ = 230 nM). Compared to the known CAi, acetazolamide, all compounds showed a good inhibition activity against *h*CAI and *h*CAII (IC_50_ < 1.3 µM), which may indicate additional therapeutic potential for these compounds [111].

## 12. Conclusions

Many merged tacrine-based, multitarget-directed AChEi have been presented throughout this review. These compounds represent various chemical scaffolds, most commonly two of the three rings of tacrine’s tricyclic core merged to other substituted heterocycles, that have been synthesized by diverse chemical methods. The Friedländer reaction has proven particularly important for building these tacrine-based inhibitors. SAR for AChE inhibition lacks a generalization across scaffolds and must be analyzed on a case-by-case basis. Nevertheless, some potent AChEi that have maintained or improved upon the already potent inhibition of tacrine have been identified, highlighted by **1s** (IC_50_ = 58 nM, 4.5-fold more potent than tacrine), **1x** (IC_50_ = 44 nM, 6-fold more potent than tacrine), **2u** (IC_50_ = 34 nM, 6-fold more potent than tacrine), **2x** (IC_50_ = 81 nM, 3-fold more potent than tacrine), **10l** (IC_50_ = 40 nM, similar potency to tacrine), **15i** (IC_50_ = 69 nM, 5-fold more potent than tacrine), **16l** (IC_50_ = 10 nM, 5.5-fold more potent than tacrine), **23l** (IC_50_ = 49 nM, 7.5-fold more potent than tacrine), **23o** (IC_50_ = 23 nM, 16-fold more potent than tacrine), **28k** (IC_50_ = 37.3 nM, 10-fold more potent than tacrine), **36c** (IC_50_ = 12.97 nM, 7-fold more potent than tacrine), **36g** (IC_50_ = 5.17 nM, 18-fold more potent than tacrine), **42o** (IC_50_ = 5.3 nM), **44a** (IC_50_ = 52 nM, similar potency to tacrine), and **54j** (IC_50_ = 37 nM, similar potency to tacrine).

A second group of compounds have been identified that either greatly reduced the hepatotoxicity of tacrine or showed significant secondary biological activity directed towards another aspect of AD (e.g., ROS, Aβ, metals), and this group is highlighted by **1f** (complete inhibition of Aβ_1-40_ *Ee*AChE-induced aggregation, neuroprotection against oligomycin A/rotenone-induced oxidative stress in cortical neurons, and reduced hepatotoxicity), **2x** (moderate inhibitor of 15-LOX and reduced hepatotoxicity), **6d** (reduced hepatotoxicity, antioxidant capacity, and neuroprotection against oligomycin/rotenone and Aβ_1-40_ in SH-SY5Ycells), **11n** (ability to chelate Cu^2+^, Zn^2+^, and Fe^2+^), **15t** (high antioxidant capacity and low hepatotoxicity), **15ac** (inhibition of *Ee*AChE-induced Aβ_1-40_ aggregation, reduced hepatotoxicity, and neuroprotection in SH-SY5Y cells against oxidative stress, Aβ_1-40_ aggregation, and tau-phosphorylation), **21** (reduced hepatotoxicity and neuroprotection in SH-SY5Y cells against ROS and tau hyperphosphorylation), **23l** (moderate ability to inhibit both self- and AChE-induced Aβ aggregation, reduced hepatotoxicity, and slight antioxidant activity), **26e** (selective COX-2 inhibition), **28k** (inhibition of Ca^2+^ influx), **42o** (antioxidant activity and significant Aβ_1-42_ self-aggregation inhibition), **44a** (antioxidant activity and reduced hepatotoxicity), and **54j** (inhibition of carbonic anhydrase). Altogether, compounds **2x**, **23l**, **28k**, **42o**, **44a**, and **54j** combine favorable AChE inhibition, favorable hepatotoxicity, and/or favorable secondary activity, and we feel they are the most promising for further study.

Going forward, we believe that continued research in this field is vital in the fight against AD. The most promising compounds identified should be further tested in animal models to evaluate if promising in vitro properties are maintained. The pharmacokinetic properties of the most promising compounds should also be further investigated. For example, the PAMPA-BBB data presented in initial screening results may be altered in an in vivo system, which would necessitate additional chemical modification to enhance pharmacokinetics. There is an additional chemical space that remains to be explored, and we envision additional heterocyclic fusions to the tacrine core to further explore SAR. As of yet, no “magic bullet” inhibitor has been identified. However, with each compound of this class that is synthesized and tested in combo, we gain incremental knowledge into the complex etiology and progression of AD that remains to be fully understood.

## Figures and Tables

**Table 1:** Inhibition of *Electrophorus electricus* (*Ee*AChE) by pyranopyrazole tacrines **1a**–**z** (data from [63,64,65]).

Cmpd	IC_50_ (nM)	Potency Index ^1^	Cmpd	IC_50_ (nM)	Potency Index ^1^
**1a**	750 ± 40	0.37	**1n**	5040 ± 1640	0.04
**1b**	590 ± 40	0.47	**1o**	1230 ± 180	0.21
**1c**	810 ± 50	0.35	**1p**	1660 ± 270	0.16
**1d**	3270 ± 190	0.09	**1q**	1800 ± 270	0.14
**1e**	310 ± 20	0.90	**1r**	170 ± 40	1.12
**1f**	1520 ± 490	0.13	**1s**	58 ± 5	4.48
**1g**	500 ± 30	0.56	**1t**	290 ± 30	0.90
**1h**	190 ± 10	1.47	**1u**	260 ± 50	1.00
**1i**	2180 ± 130	0.13	**1v**	1040 ± 480	0.25
**1j**	870 ± 60	0.32	**1w**	1770 ± 190	0.15
**1k**	320 ± 20	0.88	**1x**	44 ± 2	5.90
**1l**	720 ± 40	0.39	**1y**	330 ± 30	0.79
**1m**	5910 ± 1340	0.03	**1z**	130 ± 50	2.00

^1^ Compared to tacrine. Potency index = IC_50_ (nM) tacrine/IC_50_ (nM) cmpd. The red places emphasis on compounds highlighted in the text.

**Table 2:** Inhibition of *Ee*AChE by pyranotacrines **16a**–**t** (data from [71,78,79]).

Cmpd	IC_50_ (nM)	Potency Index ^1^	Cmpd	IC_50_ (nM)	Potency Index ^1^
**16a**	>10 µM	-	**16k**	41 ± 2	1.34
**16b**	>10 µM	-	**16l**	10 ± 1	5.50
**16c**	580 ± 140	0.33	**16m**	20 ± 3	2.75
**16d**	>10 µM	-	**16n**	90 ± 7	0.61
**16e**	2810 ± 880	0.07	**16o**	120 ± 7	0.46
**16f**	140 ± 6	0.39	**16p**	110 ± 70	0.50
**16g**	20 ± 1	2.75	**16q**	80 ± 3	0.69
**16h**	130 ± 5	0.42	**16r**	70 ± 2	0.79
**16i**	140 ± 2	0.39	**16s**	134 ± 40	0.41
**16j**	32 ± 3	1.72	**16t**	82 ± 9	0.67

^1^ Compared to tacrine. Potency index = IC_50_ (nM) tacrine/IC_50_ (nM) cmpd. The red places emphasis on compounds highlighted in the text.

**Table 3:** Inhibition of *Ee*AChE by pyrazolophthalazine tacrines **23a**–**u** (data from [85]).

Cmpd	IC_50_ (nM)	Potency Index ^1^	Cmpd	IC_50_ (nM)	Potency Index ^1^
**23a**	614	0.59	**23l**	49	7.45
**23b**	60	6.08	**23m**	3370	0.11
**23c**	80	4.56	**23n**	271	1.35
**23d**	69	5.29	**23o**	23	15.9
**23e**	100	3.65	**23p**	140	2.61
**23f**	310	1.18	**23q**	467	0.78
**23g**	592	0.62	**23r**	160	2.28
**23h**	599	0.61	**23s**	2150	0.17
**23i**	618	0.59	**23t**	1630	0.22
**23j**	193	1.89	**23u**	612	0.60
**23k**	280	1.30			

^1^ Compared to tacrine. Potency index = IC_50_ (nM) tacrine/IC_50_ (nM) cmpd. The red places emphasis on compounds highlighted in the text.

**Table 4:** Inhibition of *Ee*AChE by amidotacrines **36a**–**p** (data from [94]).

Cmpd	IC_50_ (nM)	Potency Index ^1^	Cmpd	IC_50_ (nM)	Potency Index ^1^
**36a**	-	-	**36i**	24.10 ± 3.43	3.93
**36b**	28.59 ± 3.59	3.31	**36j**	7.14 ± 0.78	13.3
**36c**	12.97 ± 0.47	7.30	**36k**	191.11 ± 10.69	0.50
**36d**	-	-	**36l**	188.70 ± 27.72	0.50
**36e**	37.16 ± 4.23	2.55	**36m**	57.64 ±9.94	1.64
**36f**	16.84 ± 2.12	5.62	**36n**	40.78 ± 5.43	2.32
**36g**	5.17 ± 0.24	18.3	**36o**	-	-
**36h**	33.74 ± 1.96	2.81	**36p**	17.72 ± 1.51	5.34

^1^ Compared to tacrine. Potency index = IC_50_ (nM) tacrine/IC_50_ (nM) cmpd. The red places emphasis on compounds highlighted in the text.

**Table 5:** Inhibition of *Ee*AChE by naphthalene pyranopyrimidinones **42a**–**i** and **43a**–**i** and naphthalene pyrimidinimines **42j**–**o** and **43j**–**o** (data from [95,96]).

Cmpd	IC_50_ (nM)	Potency Index ^1^	Cmpd	IC_50_ (nM)	Potency Index ^1^
**42a**	518.4 ± 87.9	0.09	**43a**	317.8 ± 26.0	0.14
**42b**	55.5 ± 7.1	0.80	**43b**	383.5 ± 19.4	0.12
**42c**	300.8 ± 6.5	0.15	**43c**	290.5 ± 8.3	0.15
**42d**	60.7 ± 4.5	0.73	**43d**	326.7 ± 38.9	0.14
**42e**	30.5 ± 2.8	1.45	**43e**	153.2 ± 3.1	0.29
**42f**	107.5 ± 7.2	0.41	**43f**	195.3 ± 6.2	0.23
**42g**	111.9 ± 21.7	0.40	**43g**	115.8 ± 6.2	0.38
**42h**	55.9 ± 12.7	0.79	**43h**	193.4 ± 18.7	0.23
**42i**	166.6 ± 7.8	0.27	**43i**	173.8 ± 5.9	0.25
**42j**	67.8 ± 4.3	-	**43j**	379.5 ± 12.4	-
**42k**	9.9 ± 0.5	-	**43k**	93.5 ± 6.9	-
**42l**	34.0 ± 3.9	-	**43l**	96.1 ± 2.8	-
**42m**	6.0 ± 0.1	-	**43m**	44.9 ± 1.9	-
**42n**	3.2 ± 0.2	-	**43n**	12.1 ± 0.8	-
**42o**	5.3 ± 0.2	-	**43o**	52.6 ± 0.4	-

^1^ Compared to tacrine. Potency index = IC_50_ (nM) tacrine/IC_50_ (nM) cmpd. The red places emphasis on compounds highlighted in the text.

**Table 6:** Inhibition of *Ee*AChE by indenoquinolines **54a**–**o** (data from [111]).

Cmpd	IC_50_ (nM)	Potency Index ^1^	Cmpd	IC_50_ (nM)	Potency Index ^1^
**54a**	2610 + 170	0.02	**54i**	3530 ± 117	0.02
**54b**	5700 ± 127	0.01	**54j**	37 ± 2.04	1.59
**54c**	2170 ± 273	0.03	**54k**	57 ± 2.39	1.04
**54d**	230 ± 15	0.26	**54l**	56 ± 2.49	1.05
**54e**	3730 ± 204	0.02	**54m**	4870 ± 102	0.01
**54f**	>10 µM	-	**54n**	1090 ± 175	0.05
**54g**	5410 ± 1450	0.01	**54o**	-	-
**54h**	>10 µM	-			

^1^ Compared to tacrine. Potency index = IC_50_ (nM) tacrine/IC_50_ (nM) cmpd. The red places emphasis on compounds highlighted in the text.

## Abbreviations

Aβ	Amyloid-β
ACh	Acetylcholine
AChE	Acetylcholinesterase
AChEi	Acetylcholinesterase inhibitor(s)
AD	Alzheimer’s disease
ADME	Absorption, distribution, metabolism, and excretion
APP	Amyloid precursor protein
BACE1	β-Secretase 1
BACE1i	β-Secretase 1 inhibitor(s)
BBB	Blood-brain barrier
CA	Carbonic anhydrase
CAi	Carbonic anhydrase inhibitor(s)
CAS	Catalytic active site
CNS	Central nervous system
COX-2	Cyclooxygenase 2
DABCO	1,4-Diazabicyclo[2.2.2]octane
DCE	Dichloroethane
DCM	Dichloromethane
DIPEA	*N*,*N*-Diisopropylethylamine
DMF	Dimethylformamide
DMSO	Dimethyl sulfoxide
*Ee*	*Electrophorus electricus*
GSH	Glutathione
*h*	Human
HATU	1-[Bis(dimethylamino)methylene]-1*H*-1,2,3-triazolo[4,5-*b*]pyridinium 3-oxid hexafluorophosphate
IC_50_	Half maximal inhibitory concentration
KA	Kojic acid
15-LOX	15-Lipoxygenase
MOA	Monoamine oxidase
MOAi	Monoamine oxidase inhibitor(s)
MTDL	Multitarget-directed ligand
MWI	Microwave irradiation
NFTs	Neurofibrillary tangles
NMDAR	*N*-methyl-d-aspartate receptor
ORAC	Oxygen radical absorbance capacity
PAMPA	Parallel artificial membrane permeability assay
PAS	Peripheral anionic site
*P_e_*	Effective permeability
ROS	Reactive oxygen species
SAR	Structure-activity relationship
SGPT	Serum glutamic pyruvic transaminase
*Tc*	*Torpedo californica*
TEA	Triethylamine
TFAA	Trifluoroacetic anhydride
THF	Tetrahydrofuran
T_3_P	Propylphosphonic anhydride
